# Novel C7-Substituted Coumarins as Selective Monoamine Oxidase Inhibitors: Discovery, Synthesis and Theoretical Simulation

**DOI:** 10.3390/molecules24214003

**Published:** 2019-11-05

**Authors:** Dong Wang, Ren-Yuan Hong, Mengyao Guo, Yi Liu, Nianhang Chen, Xun Li, De-Xin Kong

**Affiliations:** 1 State Key Laboratory of Agricultural Microbiology, Huazhong Agricultural University, Wuhan 430070, China; duke.e.wang@gmail.com; 2 Agricultural Bioinformatics Key Laboratory of Hubei Province, College of Informatics, Huazhong Agricultural University, Wuhan 430070, China; guomengyao96@gmail.com (M.G.); dengxue@webmail.hzau.edu.cn (Y.L.); nhchen95@gmail.com (N.C.); 3 Key Laboratory of Chemical Biology (Ministry of Education), School of Pharmaceutical Sciences, Shandong University, 44 West Culture Road, Ji’nan 250012, Shandong, China; 17865132563@163.com; 4 Institute of Materia Medica, Shandong First Medical University & Shandong Academy of Medical Sciences, No 18877, Jingshi Road, Ji’nan 250002, Shandong, China

**Keywords:** monoamine oxidase (MAO) inhibitors, BRS-3D, subtype selectivity, molecular dynamic simulations, in silico pharmacokinetic predictions

## Abstract

There is a continued need to develop new selective human monoamine oxidase (*h*MAO) inhibitors that could be beneficial for the treatment of neurological diseases. However, *h*MAOs are closely related with high sequence identity and structural similarity, which hinders the development of selective MAO inhibitors. “Three-Dimensional Biologically Relevant Spectrum (BRS-3D)” method developed by our group has demonstrated its effectiveness in subtype selectivity studies of receptor and enzyme ligands. Here, we report a series of novel C7-substituted coumarins, either synthesized or commercially purchased, which were identified as selective *h*MAO inhibitors. Most of the compounds demonstrated strong activities with IC_50_ values (half-inhibitory concentration) ranging from sub-micromolar to nanomolar. Compounds, **FR1** and **SP1**, were identified as the most selective *h*MAO-A inhibitors, with IC_50_ values of 1.5 nM (selectivity index (SI) < −2.82) and 19 nM (SI < −2.42), respectively. **FR4** and **FR5** showed the most potent *h*MAO-B inhibitory activity, with IC_50_ of 18 nM and 15 nM (SI > 2.74 and SI > 2.82). Docking calculations and molecular dynamic simulations were performed to elucidate the selectivity preference and SAR profiles.

## 1. Introduction

The dynamic stability of amines is maintained by a reuptake mechanism and also the oxidation process by monoamine oxidases (MAOs) [1]. MAO is a flavin adenine dinucleotide (FAD) containing an enzyme bound in the mitochondrial outer membrane by a transmembrane helix [2]. It is responsible for regulation of biogenic and xenobiotic amines, which includes serotonin, tryptamine, dopamine, norepinephrine, epinephrine, β-phenylethylamine, tyramine and octopamine [3]. Two MAO subtypes were identified in mammals, namely MAO-A and MAO-B. They demonstrated different tissue distribution, substrate preference and inhibitor specificity [1,4]. In addition, different *h*MAO subtypes were associated with different neurological diseases, such as depression, Alzheimer’s disease, Parkinson’s disease and Huntington’s disease. Decreases in the expression level of *h*MAO-A with age were observed, which was proved to be the causes of aggressive behavior [5]. In contrast, a four-fold increase in the *h*MAO-B expression levels with aging was thought to be an important causative factor in the etiology of neurodegenerative diseases [6]. Therefore, *h*MAO-A and *h*MAO-B were essential targets for elucidating the mechanism of monoaminergic pathways and for the treatment of psychiatric disorders and neurodegenerative diseases [7].

**Figure 1:**
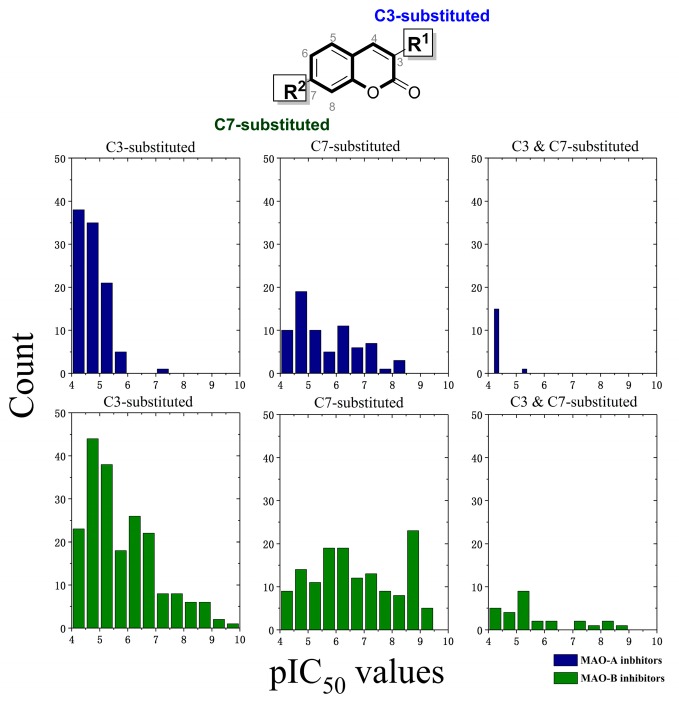
Inhibition results of C3 and C7-subsituted coumarin derivatives against *h*MAO-A and *h*MAO-B (data collected from ChEMBL v24).

Nevertheless, high sequence identity (72.6%) and structural similarity (superimposed root-mean-square deviation, RMSD = 0.94) between two *h*MAO subtypes hampered the discovery of selective MAO inhibitors (MAOIs) [4]. Efforts have been devoted for the development of subtype-selective MAO inhibitors over the last years [8,9,10]. For example, coumarin derivatives, which have many advantages such as the feasible synthesis and favorable bioavailability, were mainly designed as potential MAO inhibitors [9,11,12,13,14]. Various substitution patterns on the coumarin skeleton have been studied [14,15]. According to the orientation of pyrone ring of coumarin scaffold in the binding site, most coumarins can be categorized into two groups characterized by substitution positions: C3-substitution and C7-substitution. Of them, C3-substitution with an acyl function (e.g., carboxamide or carbohydrazide) or an aryl group, was the most promising [16]. For instance, 3-phenyl substituted coumarin derivatives displayed strong MAO-B inhibition [17,18,19] and the replacement of 3-phenyl with other heterocyclic motifs, such as thiophenyl or indolyl, also exhibited strong activity against MAO-B [9,20,21,22,23]. However, as shown in Figure 1, the development of *h*MAO-B selective inhibitors has attracted more attention than *h*MAO-A. There are very few highly potent *h*MAO-A inhibitors (pIC_50_ (−logIC_50_) > 7). As deposited in ChEMBL v24, 18 coumarins were reported to be selective to *h*MAO-B (pIC_50_ > 7 and SI > 2 (selectivity index = pIC_50_(MAO-B) − pIC_50_(MAO-A)) and only three coumarins were selective to *h*MAO-A with pIC_50_ > 7 and SI < 2 [24,25,26,27,28].

Discovery or design of subtype selective inhibitors is a challenging area due to the high sequential identity and small structural differences between the pockets of a conserved protein family (GPCRs, kinases, etc.). In such a case that protein subtypes have small structural differences, the dynamic profiles of the ligands’ conformation ensemble were believed to play an essential role in subtype selectivity. Our group recently developed a shape-similarity based protocol called “Three-Dimensional Biologically Relevant Spectrum (BRS-3D)” [29,30]. The method was validated in subtype selectivity studies of receptor and enzyme ligands [31,32,33]. A spectrum of C7-substituted coumarin analogues was discovered according to this protocol. The chemical space around these analogues were worthy to be further explored owing to their interesting structure-activity relationship (SAR) profiles. To this end, novel C7-substituted coumarin analogues were synthesized or commercially purchased and their inhibitory potencies were experimentally tested. The results revealed two C7-substituted coumarin compounds, **FR1** and **SP1**, which gave the most potent *h*MAO-A inhibitory activities, with IC_50_ values of 1.5 nM and 19 nM, respectively (SI < −2.82 and SI < −2.42). **FR4** and **FR5** showed a selective *h*MAO-B inhibitory affinity, with IC_50_ value of 18 nM and 15 nM (SI > 2.74 and SI > 2.82). In addition, docking calculations and molecular dynamics (MD) simulations were performed to a better understand this selectivity and SAR profile. In silico pharmacokinetics predictions and drug-like properties of these molecules were also evaluated.

## 2. Results and Discussion

### 2.1. Qualitative SAR of Previously Identified Hits

**Figure 2:**
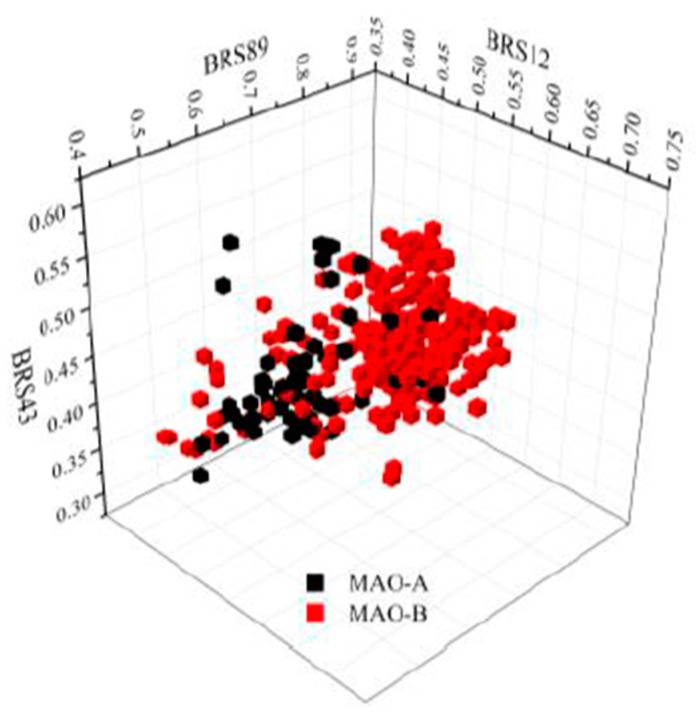
The distribution of selective MAO inhibitors on the basis of three BRS-3D features (BRS12: 1cbq_RE9, BRS43: 3i8u_18B; BRS89: 1vj5_CIU).

**Figure 3:**
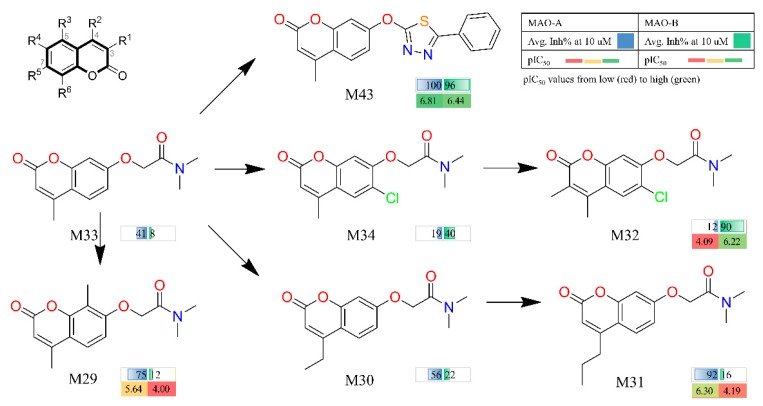
Identified coumarin analogues through BRS-3D with *h*MAO inhibitory activities.

In our previous study, BRS-3D method was introduced as a 3D shape similarity profile [29,30,32,33]. In BRS-3D, 300 diverse biologically relevant ligands were used as templates and their co-crystallized conformation was treated as the active conformation. Hence, BRS-3D was capable of reflecting the distribution of the objective compound in the known bioactive chemical space. For example, as shown in Figure 2, the different distribution of selective *h*MAO inhibitors in chemical space can be observed on the basis of three BRS-3D features (BRS12: 1cbq_RE9, BRS43: 3i8u_18B; BRS89: 1vj5_CIU). These characteristics make BRS-3D an important method for the study of subtype selectivity of receptor and enzyme ligands. Recently, we applied a BRS-3D based virtual screening protocol in the identification of selective *h*MAO inhibitors [31]. The experimental results demonstrated 70 compounds with MAO inhibition higher than 70% at a concentration of 10 μM, and 25 of them were potential hits with IC_50_ values less than 1 μM. Among the identified hits, several C7-substituted coumarin derivatives were worthy to be further explored owing to their interesting SAR profile. As illustrated in Figure 3, comparing with entries **M30**, **M31** and **M33**, R^5^ group at C7-position was fixed, and installation of extended alkyl side chains at C4-position induced more robust MAO-A inhibition (**M30**, R^2^ = ethyl, 56% vs. **M31**, R^2^ = propyl, 92%) than the methyl homologue **M33** (R^2^ = methyl, 41%). Comparing with entries **M33** and **M29**, additional introduced methyl group at C8 position gave an improved MAO-A inhibition (M29, 75% vs. **M33**, 41%). Comparing with entries **M33** and **M34**, the introduction of a Cl substitution at C6 position (**M34**) gave a nearly doubled decrease for MAO-A inhibition but a five-fold increase for MAO-B inhibition, suggesting C6-Cl substitution might be crucial to the MAO-B selectivity and binding affinity. It was also clear that the high MAO-B potency and selectivity of **M32** was largely attributable to the C6-Cl group and the additional methyl substitution. Comparing with entries **M33** and **M43**, modification of C7-substituion was clearly beneficial to both MAO-A and MAO-B inhibition. It is noteworthy that the activity of M31 and **M32** against two *h*MAO subtypes was reversed with small changes in 3-, 4- and 6-positions.

In addition, we can infer that *h*MAO-B binding site may prefer the small sized substituents at C4-position of the coumarin nucleus, and simultaneous substitutions at C3 and C4 position was beneficial to *h*MAO-B inhibition. From the SAR analysis, compound **M43** with a 2-methoxy-5-phenyl-1,3,4-thiadiazole motif at C7-position had the most potent affinity towards both MAO-A and MAO-B subtypes, which was thereby taken as a lead to optimize its activity and selectivity by fragment replacement at the 3-, 4- and 8-positions.

### 2.2. MM/PBSA Binding Energy and Decomposition Analysis

Three compounds, **M31**, **M32** and **M43**, were selected for docking simulations, with the co-crystallized ligand in 2V61 (**C18**) as reference. Based on the optimal docking models, eight systems were then investigated through MD simulations to elucidate the selectivity mechanism, four for each subtype. RMSD and RMSF (root mean square fluctuation) analysis was carried out to evaluate the structural stability. The stability of hydrogen bonds interactions between *h*MAO and C7 substituted coumarins, which are likely to contribute to the selectivity of MAOIs was also investigated. More details can be found in Supporting Information (Figures S1–S5, Table S1).

To further analyze the subtype selectivity mechanism, MM/PBSA method was employed to calculate the binding energy between *h*MAO subtypes and the discovered C7-substituted coumarins. Additionally, compound **C18** bound in 2V61, was used as a reference. The predicted binding free energy and the inhibitory activity of each compound was summarized in Table 1. The calculated binding affinities correlated well with the experimental results. For *h*MAO-A selective system, the ΔG values of MAO-A-**M31** complex and MAO-B-**M31** complex were −127.034 kJ/mol and −113.631 kJ/mol with respect to their pIC_50_ values of 6.3 and 4.2, respectively. For *h*MAO-B selective system, the opposite results were obtained. The binding affinities of MAO-A-**M32** complex and MAO-B-**M32** complex were −112.539 kJ/mol and −122.606 kJ/mol corresponding to the pIC_50_ values of 4.1 and 6.2, respectively. The MM/PBSA binding energy contained four terms: the van der Waal contributions (ΔG_vdW_), the electrostatic contributions (ΔG_ele_), the polar solvation contribution (ΔG_pol_) and the apolar solvation energy (ΔG_apol_). Then, the key terms responsible for binding were investigated separately. As listed in Table 2, taking **M31** and **M32** as an example, van der Waal contributions were the driving force for the binding in both selective systems and non-selective systems since the result of ΔG_ele_ and ΔG_pol_ was positive. This demonstrated that in *h*MAO subtypes the unspecific binding activity was mainly attributed to hydrophobic interactions while selectivity to a specific subtype was driven by the above-mentioned H-bond interactions. In addition, the electrostatic terms to the solvation free energy ΔG_pol_ were unfavorable for all systems. Compared to the polar solvation energy, the apolar solvation contributions were suggested to have a positive effect upon binding.

We then performed a residue-specific binding energy decomposition to identify key residues contributing to the subtype selectivity. The detailed inspection of per-residue energy contribution of the active site amino acids was shown in Table 3 and Table S2. Gln215 from α-helix region was calculated to be a key residue for *h*MAO-A selectivity. The energy contribution of Gln215 was −7.53 ± 0.24 kJ/mol, which is obviously larger than the contribution (−1.74 ± 0.22 kJ/mol) of the corresponding Gln206 residue in *h*MAO-B. Therefore, the decreased interaction with Gln206 reduced the activity to *h*MAO-B. Asn181 also played a critical role in *h*MAO-A selectivity since it was replaced by Cys172 in *h*MAO-B. Indeed, the corresponding energy contribution of Asn181 was −4.1478 ± 0.2633 kJ/mol for compound **M31**, which was larger than that of Cys172 in *h*MAO-B (0.2993 ± 0.124 kJ/mol). For **M32**, the Cl substitution at C6 position was thought to be crucial to *h*MAO-B selectivity. As can be seen in Table 3, the energy contribution of Cys172 (which was spatially close to C6-Cl) was −2.5912 ± 0.1582 kJ/mol, which was apparently larger than that of compound **M31** (0.2993 ± 0.124 kJ/mol). Other residues important for selectivity were also identified through the binding energy decomposition analysis. For instance, the gatekeeper residue Phe208 in *h*MAO-A and Ile199 in *h*MAO-B may contribute to the subtype selectivity. Compared with Cys323 and Leu337 in *h*MAO-A, the corresponding residue (Thr314 and Leu328) in *h*MAO-B also displayed different energy contributions, which was supported by the previous research of Catto and coworkers [34].

### 2.3. Discovery, Synthesis and Biological Activity

**Figure 4:**
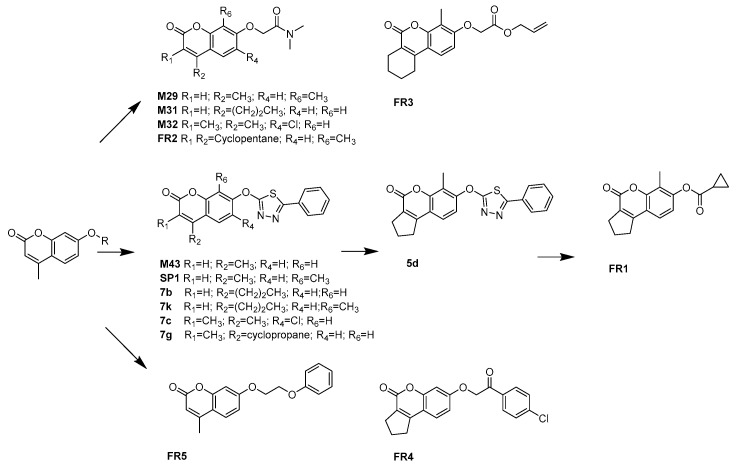
Strategy for the purchase or synthesis of C7 substituted coumarin analogues.

**Figure 5:**
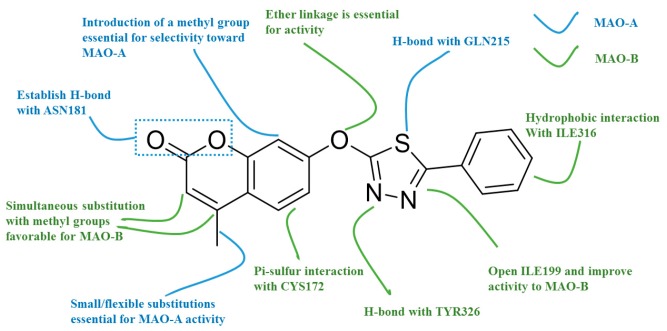
Structure-activity relationship (SAR)/ structure-selectivity relationship (SSR) profiles for the coumarins against *h*MAO subtypes based on compound **M43**.

The optimization of **M43** analogues was performed to further validate the above conclusions and explore the chemical space around the hits. This series of compounds, along with others studied in this article, were listed in Figure 4. Detailed results were listed in Table 4 (we also synthesized several Esuprone analogs, shown in Table S3). To estimate the selectivity of inhibitors, the selectivity index (SI) was calculated as pIC_50_(MAO-B)–pIC_50_(MAO-A) [14]. For **M43** derivatives, two out of six were experimentally identified as potent and selective *h*MAO-A inhibitors (**FR1**, **SP1**) with IC_50_ values of 1.5 nM (SI < –2.82) and 19 nM (SI < –2.42), respectively. Compared with **M43**, all **M43** analogues showed an improved MAO-A inhibition except compound **7c**, **7g**. From an overall SAR profiles, a direct comparison can be made between compounds **M43** and **SP1**, since the introduction of a methyl group at the C8 position in compound **M43** increased *h*MAO-A selectivity. In contrast, simply changing the substituent at C7 position of compound **M43** with other groups generally resulted in a significant decline in MAO-A selectivity (for example, compound **M43** and **FR5**). However, the replacement of R^5^ group of **5d** gave the most active *h*MAO-A inhibitors **FR1** with an IC_50_ value of 1.5 nM. In addition, comparison of the activity values of compounds **M43** and **7g** revealed the steric hindrance at C4 position again, i.e., only substituents of small size and flexibility are tolerated in *h*MAO-A inhibitors. Based on the above experimental and theoretical results, the molecular basis of the SAR and SSR (structure-selectivity relationship) trends between *h*MAO subtypes and coumarin series were summarized in Figure 5.

Compounds **M29**, **M31**, **M32** and **M43** were obtained based on our BRS-3D virtual screening protocol. **FR1**-**FR5** and **SP1** were purchased from Specs. Compounds **5d**, **7b**, **7k**, **7c** and **7g** were synthesized in this study.

### 2.4. In Silico Pharmacokinetic Properties

Additionally, in silico pharmacokinetic properties of all the tested C7-substituted coumarin derivatives were evaluated using ACD/percepta platform including lipophilicity logP, molecular weight, Lipinski’s rule of five, lead-like, Caco-2 and CNS properties. Detailed pharmacokinetic properties were presented in Table 5 [35]. According to the predicted properties, all these compounds complied with the Lipinski’s rule without any violation, which suggested that these new C7-substituted coumarin analogues might have good oral bioavailability. The Caco-2 permeability model was used to predict the absorption potential of oral drugs. The theoretical results suggested that most of these compounds possessed good intestinal permeability. A CNS penetration model was also applied suggesting the ability of these compounds to cross the blood–brain-barrier (BBB). These results suggested that the compounds might have the ability to pass the BBB, which is essential factor for MAO inhibition in human brain. In addition, a CYP isozymes inhibition model was applied for these derivatives using CypRules Server, which suggested that they do not inhibit the five CYP450 enzymes (CYP1A2, CYP2C19, CYP2C9, CYP2D6 and CYP3A4) [36].

## 3. Conclusions

An essential aspect in rational drug design or discovery against a conserved protein family is subtype selectivity. However, the small structural difference between subtypes makes it a huge challenge. In this study, based on our BRS-3D method and further SAR analysis, two selective *h*MAO-A inhibitors (**FR1**, **SP1**) and two selective *h*MAO-B inhibitors (**FR4**, **FR5**) were identified. Compound **FR1** showed the best *h*MAO-A selectivity with an IC_50_ value of 1.5 nM and SI < −2.82. **FR5** was the most active *h*MAO-B inhibitor with an IC_50_ value of 15 nM and SI > 2.82. Both docking and molecular dynamic simulations further demonstrated the details of MAO subtype selectivity at molecular level. Moreover, to evaluate drug-like properties of these molecules, *in silico* pharmacokinetic evaluation was carried out. All compounds were predicted to possess favorable pharmacokinetic profiles, and have good oral bioavailability. This analysis provided a better understanding of *h*MAO subtype selectivity mechanism using C7-subsituted coumarins as probes. In summary, the reported C7-substituted coumarins are lead compounds for developing new drugs against depression, Alzheimer’s disease and Parkinson’s disease. Furthermore, BRS-3D method mentioned in this paper can contribute to the discovery and rational design of subtype selective inhibitors for other protein families, such as GPCRs and kinases.

## 4. Materials and Methods

### 4.1. Docking Simulations

In this study, GOLD 5.2.2 program was used to dock the C7-substituted coumarin analogues into the binding site of two *h*MAO subtypes [37]. The 3D structures of C7-substituted coumarin analogues were first generated by CONCORD module and then energy minimization was performed to generate the initial conformation. The crystal structure of two *h*MAO subtypes was obtained from PDB (MAO-A: 2Z5X, MAO-B: 2V61), which were used for docking calculations. Each protein structure was optimized using the Sybyl X program in order to add hydrogen, remove the co-crystallized waters and ions. The binding area in the reference crystal structures was centered on the co-crystallized ligand with a radius of 6 Å and the Goldscore fitness function was applied to evaluate the binding affinities scores. For each ligand, 30 genetic algorithm runs were performed and the docking pose with the highest score was kept for the MD simulations.

### 4.2. Molecular Dynamic Simulations

MD simulations were performed using GROMACS 4.5.4. The AMBER ff99SB force field was selected to describe the MAO enzyme complexes [38]. The atomic parameters for the FAD cofactor were derived according to the restrained electrostatic potential (RESP) protocol at the level of HF/6-31(d) [39,40]. The force field parameters for the C7-substituted coumarins were described using the GAFF in antechamber module. The LINCS algorithm was used to keep the heavy atom H bonds in their correct lengths. Periodic boundary conditions with a 12 Å cutoff were applied in all directions for treating the non-bonded interaction. The long-range electrostatic interactions were calculated by the Particle–Mesh–Ewald (PME) method. An isothermal-isobaric ensemble (300 K, 1 atm) was employed during the MD simulation using the combination of Parrinello–Rahman method and modified Berendsen thermostat algorithm. The initial complex structure of each system was placed in an octahedral TIP3P water box. The final production run was performed for 20 ns with a restraint on the C-terminal of the protein. A time step of 2 fs was applied and the generated coordinates were saved every 100 ps.

### 4.3. MM/PBSA Calculations and Energy Decomposition Analysis

In this study, the MM/PBSA method was employed to evaluate the binding free energy between MAO subtypes and C7-subsituted coumarin analogues. The MM/PBSA binding energy was further decomposed per residue to identify key residues for subtype selectivity. The binding free energy calculations and the decomposition analysis was supported by g_mmpbsa tool [41]. For all simulated systems, the last 10 ns MD trajectory was used for analysis. In Equation (1), the first two terms, $\Delta G_{ele}\;and\;\Delta G_{vdw}$ represented electrostatic interactions and van der Waals interactions, respectively. The sum of these terms was equal to the molecular mechanics potential energy part. $\Delta G_{Polar}$ is the solvation energy contribution, which here was calculated according to the PB (Poisson Boltzmann) model. $\Delta G_{apolar}$ term represented the apolar solvation energy, which was determined based on the SASA model in this paper. As described in Equation (2), the SASA was surface-accessible solvent area, γ was set to 0.02267 kJ/mol and the fitting parameter β was set to 3.84928 kJ/mol. The last term of Equation (1), −TΔS was not considered in g_mmpbsa. Therefore, it gave the final MM/PBSA equation as presented in Equation (3).

(1) $$\Delta G_{total}=\Delta G_{ele}+\Delta G_{vdw}+\Delta G_{polar}+\Delta G_{apolar}-T\Delta S.$$

(2) $$\Delta G_{apolar}=\gamma *SASA+\beta .$$

(3) $$\Delta G_{MM/PBSA}=\Delta G_{ele}+\Delta G_{vdw}+\Delta G_{solv}.$$

### 4.4. Chemistry

**Scheme 1:**
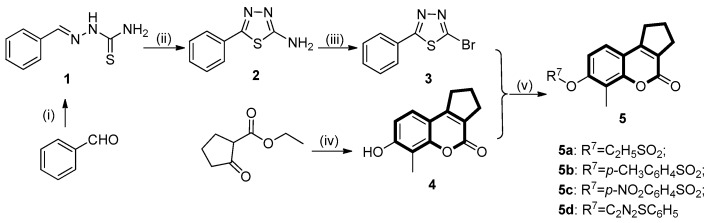
The synthetic protocol for 2,3-dihydrocyclopenta[*c*]chromen-4(1*H*)-one derivatives (**5**). Reagents and conditions: (i) Hydrazinecarbothioamide, EtOH/H_2_O, r.t.; (ii) FeCl_3_⋅6H_2_O, EtOH, 80 °C; (iii) CuBr_2_, isoamyl nitrite, CH_3_CN, r.t; (iv) Conc. H_2_SO_4_, 2-methylbenzene-1,3-diol, 0 °C and (v) K_2_CO_3_, DMF, 80 °C.

**Scheme 2:**
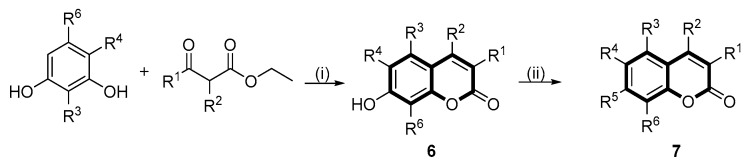
Synthetic procedures of coumarin derivatives (**7**). Reagents and conditions: (i) Method 1. H_2_SO_4_, 0 °C and (ii) 2-Bromo-5-phenyl-1,3,4-thiadiazole (compound 3), K_2_CO_3_, DMF, 80 °C. Method 2. substituted sulfuryl chloride, TEA, DCM, r.t.

The target 2,3-dihydrocyclopenta[c]chromen-4(1*H*)-one derivative (**5**) and 2*H*-chromen-2-one derivatives (**7**) were prepared according to the synthetic route outlined in Scheme 1 and Scheme 2, respectively.

#### 4.4.1. Synthesis of (E)-2-benzylidenehydrazinecarbothioamide (**1**)

Benzaldehyde (0.1 g, 0.942 mmol) was dissolved in warm anhydrous alcohol (3 mL) and a mixture of thiosemicarbazide (0.0945 g, 1.04 mmol) in warm water (3 mL) was added dropwise with continuous stirring. The mixture was allowed to react at room temperature until TLC (Thin Layer chromatography) showed the reaction has completed (PE/EA = 2:1). A large amount of white precipitate generated and filtered to give white solid (0.166 g, 0.93 mmol) yield: 98%; m.p. = 142–145 °C; ^1^H-NMR (400 MHz, DMSO-*d*_6_) δ 11.44 (s, 1H, NH), 8.28–8.14 (m, 1H, CH=N), 8.03 (d, *J* = 16.5 Hz, 2H, NH_2_), 7.80 (dd, *J* = 6.6, 2.9 Hz, 2H, ArH), 7.41 (dd, *J* = 5.2, 1.9 Hz, 3H, ArH); ESI-MS *m/z*: 180.2 [M + H]^+^.

#### 4.4.2. Synthesis of 5-phenyl-1,3,4-thiadiazol-2-amine (**2**)

Thiosemicarbazone (0.25 g, 1.39 mmol) was suspended in 5 mL of ethanol, FeCl_3_⋅6H_2_O (1.13 g, 4.18 mmol) was slowly added with constant stirring. The resulting solution was stirred for 1.5 h at 80 °C until TLC showed the reaction has completed (PE/EA = 1:1). The reaction mixture was allowed to cool to room temperature and quenched by adding citric acid (3.2 g). After neutralized with 10% ammonia solution, the crude product was precipitated and filtered to afford a white solid (0.18 g, 1.02 mmol). Yield: 72%; m.p. = 220–223 °C; ^1^H-NMR (400 MHz, DMSO-*d*_6_): δ 7.76 (dd, *J* = 7.9, 1.7 Hz, 2H, NH_2_), 7.53–7.36 (m, 5H, ArH); ESI-MS *m/z*: 175.8 [M + H]^−^.

#### 4.4.3. Preparation of 2-bromo-5-phenyl-1,3,4-thiadiazole (**3**)

The copper bromide (0.116 g, 0.520 mmol) and isoamyl nitrite (0.061 g, 0.520 mmol) were added successively in CH_3_CN (10 mL). The resulting mixture was stirred at room temperature for 10 min, then 5-phenyl-1,3,4-thiadiazol-2-amine (**2**, 0.04 g, 0.226 mmol) was added in one portion. The reaction mixture was allowed to react at 25 °C for 1 h until completion, as determined by TLC. The solvent was subsequently removed under reduced pressure, and the residue was washed with ethyl acetate (3 × 20 mL). The organic phase was extracted successively with 2N HCl (3 × 25 mL) and brine (25 mL). The combined organic phase was dried over MgSO_4_ and filtered. All the solvent was removed under vacuum and the resulting residue was purified by flash column chromatography (PE/EA = 1:1, *v:v*) to afford light yellow solid (0.049 g, 0.20 mmol). Yield: 90.7%; m.p. = 82–84 °C; ^1^H-NMR (400 MHz, DMSO-*d*_6_): δ 8.10–7.77 (m, 2H, ArH), 7.71–7.44 (m, 3H, ArH); ^13^C-NMR (100 MHz, CDCl_3_): δ 172.16, 137.99, 131.79, 129.37, 127.85; ESI-MS *m/z*: 240.7 [M + H]^+^.

#### 4.4.4. General Procedure for the Synthesis of 7-hydroxy-6-methyl-2,3-dihydrocyclopenta[c]chromen- 4(1H)-one (**4**)

To a solution of 2-methylbenzene-1,3-diol (0.1 g) in alkylated ethyl acetoacetate, concentrated sulfuric acid (1 mL) was slowly added dropwise at 0 °C in an ice bath. The resulting solution was allowed to react at 0 °C for 5–6 h until the disappearance of starting material, as monitored by TLC (MeOH/DCM = 100:1). The reaction was quenched by adding 50 mL of ice water. The obtained crude solid was filtered, washed with water, dried, and recrystallized by 95% ethanol to give the desired compound (4) as a light yellow solid. Yield: 78%; m.p. = 243–244 °C; ^1^H-NMR (400 MHz, DMSO-*d*_6_): δ 10.32 (s, 1H, OH), 7.25 (d, *J* = 8.0 Hz, 1H, ArH), 6.84 (d, *J* = 8.0 Hz, 1H, ArH), 3.00 (t, *J* = 6.9 Hz, 2H, CH_2_CH_2_CH_2_), 2.71 (t, *J* = 6.8 Hz, 2H, CH_2_CH_2_CH_2_), 2.16 (s, 3H, CH_3_), 2.08–2.06 (m, 2H, CH_2_CH_2_CH_2_); ESI-MS *m/z*: 215.8 [M + H]^−^.

#### 4.4.5. Preparation of 7-hydroxyl Substituted Coumarin Derivatives (**6a–e**)

The 7-hydroxyl substituted coumarin derivatives (**6a–e**) were accomplished according to the similar procedures as that of compound 4.

*1Hydroxy-4-methyl-2H-chromen-2-one* (**6a**). Light yellow solid, yield: 56%; m.p. = 180–182 °C; ^1^H-NMR (400 MHz, DMSO-*d*_6_): δ 10.53 (s, 1H, OH), 7.60 (d, *J* = 8.7 Hz, 1H, ArH), 6.81 (dd, *J* = 8.7, 2.4 Hz, 1H, ArH), 6.71 (d, *J* = 2.4 Hz, 1H, ArH), 6.13 (d, *J* = 1.4 Hz, 1H, C=CH), 2.37 (s, 3H, CH_3_); ESI-MS *m/z*: 174.5 [M + H]^−^.

*7-Hydroxy-4-propyl-2H-chromen-2-one* (**6b**). Light yellow solid, yield: 76.6%; m.p. = 128–130 °C; ^1^H-NMR (400 MHz, DMSO-*d*_6_) δ 10.55 (s, 1H, OH), 7.81–7.11 (m, 1H, ArH), 6.91–6.57 (m, 2H, ArH), 6.08 (s, 1H, C=CH), 2.71 (t, *J* = 7.8 Hz, 2H, CH_2_CH_2_CH_3_), 1.78–1.46 (m, 2H, CH_2_CH_2_CH_3_), 1.12–0.79 (m, 3H, CH_2_CH_2_CH_3_); ESI-MS *m/z*: 203 [M + H]^−.^

*6-Chloro-7-hydroxy-3,4-dimethyl-2H-chromen-2-one* (**6c**). Light yellow solid, yield: 81.1%; m.p. = 258–260 °C; ^1^H-NMR (400 MHz, DMSO-*d*_6_): δ 11.19 (s, 1H, OH), 7.75 (s, 1H, ArH), 6.87 (s, 1H, ArH), 2.34 (s, 3H, CH_3_), 2.06 (s, 3H, CH_3_); ESI-MS *m/z*: 225 [M + H]^+^.

*4-Cyclopropyl-7-hydroxy-2H-chromen-2-one* (**6d**). Light yellow solid, yield: 86%; m.p. = 199–200 °C; ^1^H-NMR (400 MHz, DMSO-*d*_6_): δ 10.54 (s, 1H, OH), 7.90 (d, *J* = 8.7 Hz, 1H, ArH), 6.83 (dd, *J* = 8.7, 2.4 Hz, 1H, ArH), 6.71 (d, *J* = 2.4 Hz, 1H, ArH), 5.82 (s, 1H, C=CH), 2.24 (dd, *J* = 8.5, 4.2 Hz, 1H, CHCH_2_CH_2_), 1.17–1.00 (m, 2H, CHCH_2_CH_2_), 0.98–0.76 (m, 2H, CHCH_2_CH_2_); ESI-MS *m/z*: 202.9 [M + H]^+^.

*7-Hydroxy-8-methyl-4-propyl-2H-chromen-2-one* (**6e**). Light yellow solid, yield: 72%; m.p. = 160 °C; ^1^H-NMR (400 MHz, DMSO-*d*_6_): δ 10.42 (s, 1H, OH), 7.50 (d, *J* = 8.7 Hz, 1H, ArH), 6.86 (d, *J* = 8.7 Hz, 1H, ArH), 6.08 (s, 1H, C=CH), 2.70 (t, *J* = 7.6 Hz, 2H, CH_2_CH_2_CH_3_), 2.15 (s, 3H, CH_3_), 1.67–1.62 (m, 2H, CH_2_CH_2_CH_3_), 0.96 (t, *J* = 7.3 Hz, 3H, CH_2_CH_2_CH_3_); ESI-MS *m/z*: 217.8 [M + H]^−^.

#### 4.4.6. General Procedure for the Synthesis of Target Compounds (**5d**, **7a**–**c**, **7g** and **7k**)

Appropriate coumarin analogues (0.5 mmol) and 2-bromo-5-phenyl-1,3,4-thiadiazole (3, 0.8 mmol) were dissolved in anhydrous DMF (10 mL), and K_2_CO_3_ (2.0 mmol) was added in one portion. The mixture was then stirred at 100 °C for 6–7 h under an inert nitrogen atmosphere. Once the completion of the reaction was detected by TLC, the reaction mixture was cooled to room temperature. The reaction mixture was extracted with ethyl acetate (3 × 20 mL) and the combined organic layer was washed with brine (3 × 25 mL) and dried over anhydrous sodium sulfate and filtered. The solvent was removed under vacuum and the crude product was purified by flash chromatography (silica gel, PE/EA = 10:3) to afford the compound **5d** as white powder. Yield: 56%; m.p. = 140 °C; ^1^H-NMR (400 MHz, DMSO-*d*_6_): δ 7.92–7.83 (m, 2H, ArH), 7.62 (d, *J* = 8.6 Hz, 1H, ArH), 7.58–7.47 (m, 4H, ArH), 3.13 (t, *J* = 7.7 Hz, 2H,CH_2_CH_2_CH_2_), 2.81 (t, *J* = 7.6 Hz, 2H, CH_2_CH_2_CH_2_), 2.34 (s, 3H, CH_3_), 2.23–1.99 (m, 2H, CH_2_CH_2_CH_2_); ^13^C-NMR (100 MHz, CDCl_3_): δ 173.55, 164.27, 159.83, 155.91, 155.03, 153.43, 131.11, 130.32, 129.17, 127.51, 127.21, 123.00, 118.71, 117.01, 116.18, 32.20, 30.62, 22.51, 9.38; ESI-MS *m/z*: 377.0 [M + H]^+^.

*4-Methyl-7-((5-phenyl-1,3,4-thiadiazol-2-yl)oxy)-2H-chromen-2-one* (**7a**). White powder, yield: 42.8%; m.p. = 124–125 °C; ^1^H-NMR (400 MHz, DMSO-*d*_6_): δ 7.94–7.90 (m, 3H), 7.66 (d, *J* = 2.5 Hz, 1H, ArH), 7.62–7.47 (m, 4H, ArH), 6.45 (d, *J* = 1.4 Hz, 1H, C=CH), 2.47 (s, 3H, CH_3_); ^13^C-NMR (100 MHz, CDCl_3_): δ 172.31, 164.90, 160.29, 157.08, 154.48, 151.76, 131.27, 130.19, 129.22, 127.31, 126.06, 117.93, 115.67, 114.66, 108.16, 18.81; ESI-MS *m/z*: 336.9 [M + H]^+^.

*7-((5-Phenyl-1,3,4-thiadiazol-2-yl)oxy)-4-propyl-2H-chromen-2-one* (**7b**). White powder, yield: 48.3%; m.p. = 150 °C; ^1^H-NMR (400 MHz, DMSO-*d*_6_): δ 7.99 (d, *J* = 8.8 Hz, 1H, ArH), 7.95–7.88 (m, 2H, ArH), 7.66 (d, *J* = 2.5 Hz, 1H, ArH), 7.60–7.53 (m, 3H, ArH), 7.50 (dd, *J* = 8.8, 2.5 Hz, 1H, ArH), 6.39 (s, 1H, C=CH), 2.82 (t, *J* = 7.6 Hz, 2H, CH_2_CH_2_CH_3_), 1.72–1.63 (m, 2H, CH_2_CH_2_CH_3_), 1.02 (t, *J* = 7.3 Hz, 3H, CH_2_CH_2_CH_3_); ^13^C-NMR (100 MHz, CDCl_3_): δ 172.34, 164.88, 160.55, 156.91, 155.47, 154.71, 131.27, 130.37, 129.22, 127.31, 125.84, 117.31, 115.64, 113.52, 108.36, 33.81, 21.30, 13.94; ESI-MS *m/z*: 364.9 [M + H]^+^.

*6-Chloro-3,4-dimethyl-7-((5-phenyl-1,3,4-thiadiazol-2-yl)oxy)-2H-chromen-2-one* (**7c**). White powder, yield: 51.3%; m.p. = 171–173 °C; ^1^H-NMR (400 MHz, DMSO-*d*_6_): δ 8.12 (s, 1H, ArH), 7.94–7.88 (m, 3H, ArH), 7.60–7.52 (m, 3H, ArH), 2.45 (s, 3H, CH_3_), 2.15 (s, 3H, CH_3_); ^13^C-NMR (100 MHz, CDCl_3_): δ 172.32, 164.93, 161.01, 151.21, 150.98, 144.32, 131.24, 130.19, 129.20, 127.29, 126.07, 123.37, 121.59, 119.96, 110.64, 15.32, 13.67; ESI-MS *m/z*: 385.0 [M + H]^+^.

*4-Cyclopropyl-7-((5-phenyl-1,3,4-thiadiazol-2-yl)oxy)-2H-chromen-2-one* (**7g**).White powder, yield: 89%; m.p. = 158–160 °C; ^1^H-NMR (400 MHz, CDCl_3_): δ 7.99 (d, *J* = 8.6 Hz, 1H, ArH), 7.87 (dd, *J* = 7.5, 2.2 Hz, 2H, ArH), 7.54–7.44 (m, 3H, ArH), 7.43–7.35 (m, 2H, ArH), 6.06 (s, 1H, C=CH), 2.14–1.97 (m, 1H, CH), 1.22–1.11 (m, 2H, CH_2_CH_2_), 0.94–0.80 (m, 2H, CH_2_CH_2_); ^13^C-NMR (100 MHz, CDCl_3_): δ 172.36, 165.43, 160.91, 157.07, 157.06, 154.36, 131.24, 130.22, 129.21, 127.31, 126.24, 118.21, 115.61, 110.05, 108.14, 12.17, 8.07; ESI-MS *m/z*: 362.8 [M + H]^+^.

*8-Methyl-7-((5-phenyl-1,3,4-thiadiazol-2-yl)oxy)-4-propyl-2H-chromen-2-one* (**7k**). White powder, yield: 46.8%; m.p. = 110–112 °C; ^1^H-NMR (400 MHz, DMSO-*d*_6_): δ 7.92–7.86 (m, 2H, ArH), 7.84 (d, *J* = 8.9 Hz, 1H, ArH), 7.60–7.52 (m, 3H, ArH), 7.49 (d, *J* = 8.8 Hz, 1H, ArH), 6.40 (s, 1H, C=CH), 2.81 (t, *J* = 7.6 Hz, 2H, CH_2_CH_2_CH_3_), 2.32 (s, 3H, CH_3_), 1.67 (q, *J* = 7.5 Hz, 2H, CH_2_CH_2_CH_3_), 1.00 (t, *J* = 7.3 Hz, 3H, CH_2_CH_2_CH_3_); ^13^C-NMR (100 MHz, CDCl_3_): δ 173.28, 164.40, 160.75, 155.89, 155.41, 153.13, 131.15, 130.28, 129.18, 127.23, 122.69, 118.95, 117.51, 116.05, 113.41, 33.93, 21.41, 13.94, 9.25; ESI-MS *m/z*: 377.0 [M + H]^−^.

#### 4.4.7. Synthesis of the Coumarin Sulfonates (**5a**–**c**, **7d**–**f** and **7h**–**j**)

A solution of the appropriate coumarin analogues (0.1 mmol) and triethylamine (0.2 mmol) in dry dichloromethane (5 mL) was cooled in an ice bath. A solution of an appropriate sulfonyl chloride derivative (0.12 mmol) in dry dichloromethane (2 mL) was subsequently added dropwise at the same temperature. The reaction mixture was allowed to warm to room temperature and stirred for additional 0.5 h until the disappearance of starting material, as monitored by TLC. The reaction mixture was extracted with ethyl acetate (3 × 20 mL), and the combined organic phase was washed with brine (3 × 25 mL) and dried over anhydrous sodium sulfate. The solvent was evaporated under reduced pressure and the crude residue was purified by flash column chromatography (silica gel, PE/EA = 10:3 to 10:4, *v:v*) to afford the desired compounds.

6-Methyl-4-oxo-1,2,3,4-tetrahydrocyclopenta[c]chromen-7-yl ethanesulfonate (**5a**). White powder. Yield: 86%; m.p. = 150–151 °C; ^1^H-NMR (400 MHz, DMSO-*d*_6_) δ 7.55 (d, *J* = 8.6 Hz, 1H, ArH), 7.34 (d, *J* = 8.7 Hz, 1H, ArH), 3.71 (q, *J* = 7.3 Hz, 2H, CH_3_CH_2_), 3.10 (t, *J* = 8.4 Hz,2H, CH_2_CH_2_CH_2_), 2.79 (t, *J* = 7.4 Hz, 2H, CH_2_CH_2_CH_2_), 2.35 (s, 3H, CH_3_), 2.18–2.09 (m, 2H, CH_2_CH_2_CH_2_), 1.43 (t, *J* = 7.3 Hz, 3H, CH_3_CH_2_); ^13^C-NMR (100 MHz, CDCl_3_): δ 159.66, 155.75, 153.24, 148.50, 127.81, 122.56, 120.51, 118.16, 117.39, 46.37, 32.15, 30.62, 22.47, 9.89, 8.35; ESI-MS *m/z*: 309.4 [M + H]^+^.

*6-Methyl-4-oxo-1,2,3,4-tetrahydrocyclopenta[c]chromen-7-yl 4-methylbenzenesulfonate* (**5b**). White powder, yield: 81%; m.p. = 152 °C; ^1^H-NMR (400 MHz, CDCl_3_): δ 7.73 (d, *J* = 8.2 Hz, 2H, ArH), 7.33 (d, *J* = 8.1 Hz, 2H, ArH), 7.27 (d, *J* = 3.5 Hz, 1H, ArH), 7.13 (d, *J* = 8.6 Hz, 1H, ArH), 3.06 (dd, *J* = 8.2, 1.9 Hz, 2H, CH_2_CH_2_CH_2_), 3.00–2.78 (m, 2H, CH_2_CH_2_CH_2_), 2.47 (s, 3H, CH_3_), 2.27–2.14 (m, 2H, CH_2_CH_2_CH_2_), 2.07 (s, 3H, CH_3_); ^13^C-NMR (100 MHz, CDCl_3_): δ 159.75, 155.84, 153.00, 149.32, 145.89, 132.56, 130.01, 128.39, 127.77, 122.38, 120.67, 118.61, 117.29, 32.15, 30.59, 22.48, 21.80, 9.49; ESI-MS *m/z*: 370.8 [M + H]^+^.

*6-Methyl-4-oxo-1,2,3,4-tetrahydrocyclopenta[c]chromen-7-yl 4-nitrobenzenesulfonate* (**5c**). White powder, yield: 79%; m.p. = 192–194 °C; ^1^H-NMR (400 MHz, DMSO-*d*_6_): δ 8.59–8.36 (m, 2H, ArH), 8.31–8.08 (m, 2H, ArH), 7.51 (d, *J* = 8.6 Hz, 1H, ArH), 7.07 (d, *J* = 8.6 Hz, 1H, ArH), 3.07 (t, *J* = 7.7 Hz, 2H, CH_2_CH_2_CH_2_), 2.77 (t, *J* = 7.5 Hz, 2H, CH_2_CH_2_CH_2_), 2.13 (t, *J* = 7.6 Hz, 2H, CH_2_CH_2_CH_2_), 2.08 (s, 3H, CH_3_); ^13^C-NMR (100 MHz, DMSO-*d*_6_): δ 158.87, 156.38, 152.87, 151.76, 148.68, 140.03, 130.47, 128.08, 125.72, 124.24, 119.84, 118.50, 117.84, 32.21, 30.71, 22.43, 9.91; ESI-MS *m/z*: 402.1 [M + H]^+^.

*4-Cyclopropyl-2-oxo-2H-chromen-7-yl ethanesulfonate* (**7d**). White powder, yield: 81%; m.p. = 92 °C; ^1^H-NMR (400 MHz, DMSO-*d*_6_): δ 8.19 (d, *J* = 8.8 Hz, 1H, ArH), 6.16 (s, 1H, C=CH), 3.63 (q, *J* = 7.3 Hz, 2H, CH_3_CH_2_), 2.35–2.29 (m, 1H, CH), 1.40 (t, *J* = 7.3 Hz, 3H, CH_3_CH_2_), 1.11 (dt, *J* = 8.3, 3.3 Hz, 2H, CH_2_CH_2_), 0.99–0.84 (m, 2H, CH_2_CH_2_); ^13^C-NMR (100 MHz, CDCl_3_): δ 160.63, 156.90, 154.07, 150.87, 126.19, 119.21, 118.19, 110.69, 110.65, 45.76, 12.17, 8.28, 8.03; ESI-MS *m/z*: 294.7 [M + H]^+^.

*4-Cyclopropyl-2-oxo-2H-chromen-7-yl 4-methylbenzenesulfonate* (**7e**). White powder, yield: 86%; m.p. = 132–134 °C; ^1^H-NMR (400 MHz, DMSO-*d*_6_): δ 8.10 (d, *J* = 8.8 Hz, 1H, ArH), 7.88–7.68 (m, 2H, ArH), 7.50 (d, *J* = 8.1 Hz, 2H, ArH), 7.14 (d, *J* = 2.3 Hz, 1H, ArH), 7.09 (dd, *J* = 8.8, 2.4 Hz, 1H, ArH), 6.12 (s, 1H, C=CH), 2.44 (s, 4H), 2.32–2.12 (m, 1H, CH), 1.08 (dt, *J* = 8.3, 3.3 Hz, 2H, CH_2_CH_2_), 0.92–0.88 (m, 2H, CH_2_CH_2_); ^13^C-NMR (100 MHz, CDCl_3_): δ 160.72, 157.01, 153.73, 151.52, 146.03, 132.22, 130.07, 128.47, 125.91, 119.12, 118.82, 110.88, 110.48, 21.81, 12.13, 8.10; ESI-MS *m/z*: 356.7 [M + H]^+^.

*4-Cyclopropyl-2-oxo-2H-chromen-7-yl 4-nitrobenzenesulfonate* (**7f**). White powder, yield: 88%; m.p. = 192–194 °C; ^1^H-NMR (400 MHz, DMSO-*d*_6_): δ 8.59–8.39 (m, 2H, ArH), 8.31–8.18 (m, 2H, ArH), 8.12 (d, *J* = 8.8 Hz, 1H, ArH), 7.26 (d, *J* = 2.4 Hz, 1H, ArH), 7.16 (dd, *J* = 8.8, 2.4 Hz, 1H, ArH), 6.14 (s, 1H, C=CH), 2.33–2.16 (m, 1H, CH), 1.09 (dt, *J* = 8.3, 3.2 Hz, 2H, CH_2_CH_2_), 0.91 (dt, *J* = 6.8, 3.4 Hz, CH_2_CH_2_); ^13^C-NMR (100 MHz, CDCl_3_): δ 160.32, 156.70, 153.90, 151.23, 150.78, 140.61, 129.88, 126.27, 124.63, 119.67, 118.14, 111.01, 110.80, 12.15, 8.10; ESI-MS *m/z*: 388.0 [M+H]^+^.

*8-Methyl-2-oxo-4-propyl-2H-chromen-7-yl ethanesulfonate* (**7h**). White powder, yield: 71%; m.p. = 78–80 °C; ^1^H-NMR (400 MHz, DMSO-*d*_6_): δ 7.79 (d, *J* = 8.8 Hz, 1H, ArH), 7.35 (d, *J* = 8.8 Hz, 1H, ArH), 6.39 (s, 1H, C=CH), 3.72 (q, *J* = 7.3 Hz, 2H, CH_3_CH_2_), 2.79 (t, *J* = 7.6 Hz, 2H, CH_2_CH_2_CH_3_), 2.33 (s, 3H, CH_3_), 1.70–1.61 (m, 2H, CH_2_CH_2_CH_3_), 1.43 (t, *J* = 7.3 Hz, 3H, CH_3_CH_2_), 0.99 (t, *J* = 7.3 Hz, 3H, CH_2_CH_2_CH_3_); ^13^C-NMR (100 MHz, CDCl_3_): δ 160.55, 155.75, 152.95, 149.03, 122.30, 120.79, 118.05, 117.95, 113.68, 46.42, 33.87, 21.38, 13.89, 9.74, 8.34; ESI-MS *m/z*: 310.7[M + H]^+^.

*8-Methyl-2-oxo-4-propyl-2H-chromen-7-yl 4-methylbenzenesulfonate* (**7i**). White powder, yield: 83%; m.p. = 144–146 °C; ^1^H-NMR (400 MHz, DMSO-*d*_6_): δ 7.81 (d, *J* = 8.2 Hz, 2H, ArH), 7.75 (d, *J* = 8.9 Hz, 1H, ArH), 7.52 (d, *J* = 8.1 Hz, 2H, ArH), 7.03 (d, *J* = 8.8 Hz, 1H, ArH), 6.37 (s, 1H, C=CH), 2.76 (t, *J* = 7.6 Hz, 2H, CH_2_CH_2_CH_3_), 2.45 (s, 3H, CH_3_), 2.01 (s, 3H, CH_3_), 1.66–1.60 (m, 2H, CH_2_CH_2_CH_3_), 0.97 (t, *J* = 7.3 Hz, 3H, CH_2_CH_2_CH_3_); ^13^C-NMR (100 MHz, CDCl_3_): δ 160.61, 155.77, 152.73, 149.87, 145.92, 132.66, 130.04, 128.37, 122.06, 120.97, 118.47, 117.86, 113.61, 33.86, 21.79, 21.34, 13.91, 9.35; ESI-MS *m/z*: 372.8 [M + H]^+^.

*8-Methyl-2-oxo-4-propyl-2H-chromen-7-yl 4-nitrobenzenesulfonate* (**7j**). White powder, yield: 86%; m.p. = 171–174 °C; ^1^H-NMR (400 MHz, DMSO-*d*_6_): δ 8.49 (d, *J* = 8.7 Hz, 2H, ArH), 8.23 (d, *J* = 8.6 Hz, 2H, ArH), 7.75 (d, *J* = 8.9 Hz, 1H, ArH), 7.07 (d, *J* = 8.8 Hz, 1H, ArH), 6.40 (s, 1H, C=CH), 2.77 (t, *J* = 7.6 Hz, 2H, CH_2_CH_2_CH_3_), 2.07 (s, 3H, CH_3_), 1.68–1.61 (m, 2H, CH_2_CH_2_CH_3_), 0.98 (t, *J* = 7.3 Hz, 3H, CH_2_CH_2_CH_3_); ^13^C-NMR (100 MHz, CDCl_3_): δ 160.22, 155.50, 152.92, 151.21, 149.28, 141.33, 129.78, 124.63, 122.40, 120.90, 118.38, 117.79, 114.06, 33.84, 21.32, 13.90, 9.69; ESI-MS *m/z*: 402.0 [M + H]^−^.

### 4.5. Biological Assays

The inhibition and IC_50_ values of the identified C7-substituted coumarin analogues were assayed by Shanghai ChemPartner Co. Ltd., using MAO-GloTM assay kit (Promega Corporation, V1402). Two human MAO enzymes (Cat#31502, Cat#31503) were purchased from Active Motif. Clorgyline and R(-)-deprenyl were purchased as reference drugs from Sigma (Cat. No. M3778) and Abcam (Cat. No. ab120604), respectively. The MAO inhibition assays were carried out in modified HEPES buffer in light of the manufacturer’s protocol. Firstly, the tested compounds in DMSO were transferred to a 384-well plate by Echo. The enzyme solutions were then added into the plate and the plate was incubated for 15 min at room temperature (r.t.). Secondly, the luciferin derivative substrate was added to initiate the reaction. After 60 min incubation at r.t., the Luciferin detection reagent was added to each well to terminate the reaction and generate the luminescent signal. Before reading the signal, the plate was incubated for 20 min at r.t. Finally, plate reader was used to measure and record the luminescent signal and corresponding data were processed using GraphPad Prism 5.

## Figures and Tables

**Table 1:** Experimental activities and MM/PBSA binding free energies.

	MAO-A			MAO-B		
Cmpd	Inh%	pIC_50_	binding free energy	Inh%	pIC_50_	binding free energy
**M29**	75	5.6	−121.03 ± 0.92	12	4	−109.72 ± 1.07
**M30**	56	ND	−116.90 ± 1.09	22	ND	−102.79 ± 1.27
**M31**	92	6.3	−127.03 ± 1.28	16	4.2	−113.63 ± 1.26
**M32**	12	4.1	−112.54 ± 1.12	90	6.2	−122.61 ± 1.12
**M33**	41	ND	−115.25 ± 1.01	8	ND	−105.59 ± 1.08
**M34**	19	ND	−107.67 ± 0.99	40	ND	−111.18 ± 0.95
**M43 (7a)**	102	6.8	−129.34 ± 1.10	96	6.4	−135.59 ± 1.11
**C18**	-	4.8	−96.05 ± 1.31	-	7	−144.57 ± 1.29

*ND: the IC_50_ values were not determined due to the low inhibitory activity. pIC_50_ = −log(IC_50_).

**Table 2:** Analysis of calculated binding free energies of four systems.

Terms	MAO-A vs. M31	MAO-B vs. M31	MAO-A vs. M32	MAO-B vs. M32
ΔG_vdW_	−211.794 ± 0.990	−198.535 ± 1.147	−214.799 ± 0.969	−198.570 ± 1.187
ΔG_ele_	−38.523 ± 0.817	−61.460 ± 0.704	−31.376 ± 0.547	−1.362 ± 0.727
ΔG_pol_	140.734 ± 0.539	164.645 ± 1.247	151.326 ± 0.787	144.800 ± 0.895
ΔG_nonpol_	−17.431 ± 0.071	−18.241 ± 0.073	−17.715 ± 0.066	−17.418 ± 0.065
ΔG	−127.034 ± 1.281	−113.631 ± 1.260	−12.539 ± 1.116	−122.598 ± 1.144

**Table 3:** Free energy contributions (kJ/mol) of *h*MAO-A and *h*MAO-B pocket residues.

Residue		MAO-A vs. M31	MAO-B vs. M31	MAO-A vs. M32	MAO-B vs. M32
**MAO-A**	MAO-B	Total Energy	Total Energy	Total Energy	Total Energy
**Tyr69**	Tyr60	−1.4631 ± 0.0443	−3.5967 ± 0.0614	−2.7071 ± 0.0511	−3.0942 ± 0.0708
**Ala111**	Pro102	0.3332 ± 0.0714	0.8449 ± 0.1369	0.5828 ± 0.0985	−0.0127 ± 0.0890
**Phe112**	Phe103	−0.3019 ± 0.0205	0.4229 ± 0.0305	−0.1243 ± 0.0153	0.0942 ± 0.0165
**Pro113**	Pro104	−0.0764 ± 0.0091	−0.0264 ± 0.0079	0.0414 ± 0.0055	−0.0514 ± 0.0163
**Trp128**	Trp119	−0.0145 ± 0.0064	−0.0097 ± 0.0097	0.0893 ± 0.0033	−0.1936 ± 0.0077
**Phe173**	Leu164	−0.0364 ± 0.0108	−0.6217 ± 0.0187	−0.0662 ± 0.0114	−0.4042 ± 0.0134
**Leu176**	Leu167	−0.0701 ± 0.0213	−0.0436 ± 0.0256	0.1918 ± 0.0137	−0.2503 ± 0.0171
**Phe177**	Phe168	−0.1931 ± 0.0494	−0.4662 ± 0.026	0.6680 ± 0.0293	−0.5791 ± 0.0819
**Ile180**	Leu171	−5.3303 ± 0.1123	−6.2815 ± 0.1427	−2.4365 ± 0.2223	−7.2863 ± 0.1543
**Asn181**	Cys172	−4.1478 ± 0.2633	0.2993 ± 0.124	0.8583 ± 0.2391	−2.5912 ± 0.1582
**Tyr197**	Tyr188	1.1053 ± 0.0829	1.7647 ± 0.076	−4.1025 ± 0.1387	0.1502 ± 0.0949
**Ile207**	Ile198	−3.9048 ± 0.1647	−0.9873 ± 0.1489	−3.1187 ± 0.1347	−1.9079 ± 0.1410
**Phe208**	Ile199	−6.1625 ± 0.2403	−1.1994 ± 0.2342	−2.2755 ± 0.2184	0.0638 ± 0.1966
**Gln215**	Gln206	−7.5319 ± 0.236	−1.7379 ± 0.2144	−3.3143 ± 0.2539	−1.3530 ± 0.2173
**Cys323**	Thr314	−0.5525 ± 0.0492	−2.5552 ± 0.0515	−0.4392 ± 0.0685	−2.3485 ± 0.0513
**Ile325**	Ile316	−1.7599 ± 0.0637	−3.1339 ± 0.0787	−1.6549 ± 0.0398	−4.0400 ± 0.0843
**Ile335**	Tyr326	−4.5914 ± 0.1095	−4.8286 ± 0.25	−4.2492 ± 0.1381	−5.9530 ± 0.1767
**Thr336**	Thr327	−0.4413 ± 0.0476	−0.3823 ± 0.0269	−0.0784 ± 0.0246	−0.5360 ± 0.0223
**Leu337**	Leu328	−3.0775 ± 0.0836	−1.0562 ± 0.026	−2.2824 ± 0.0758	−1.1358 ± 0.0269
**Met350**	Met341	−0.168 ± 0.0143	−0.438 ± 0.0118	−0.6131 ± 0.0173	−0.3265 ± 0.0107
**Phe352**	Phe343	−0.501 ± 0.0391	−2.0697 ± 0.1135	−1.2125 ± 0.0482	−2.5305 ± 0.0643
**Tyr407**	Tyr398	−5.3629 ± 0.1462	−6.0795 ± 0.1569	−7.7790 ± 0.1624	−8.5574 ± 0.1847
**Tyr444**	Tyr435	−3.919 ± 0.2074	−2.1508 ± 0.1615	−7.4496 ± 0.1530	−1.9394 ± 0.1860

**Table 4:**
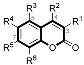
In vitro activities against *h*MAO subtypes of coumarin derivatives.

Compounds	R^1^	R^2^	R^4^	R^5^	R^6^	MAO-A ^a^	MAO-B ^a^	SI ^b^
**M29**	H	CH_3_	H	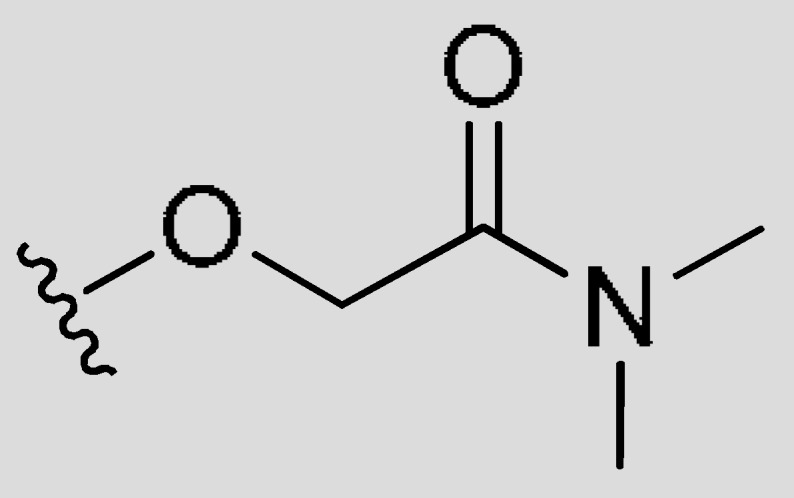	CH_3_	2.29	100	−1.64
**M31**	H	(CH_2_)_2_CH_3_	H	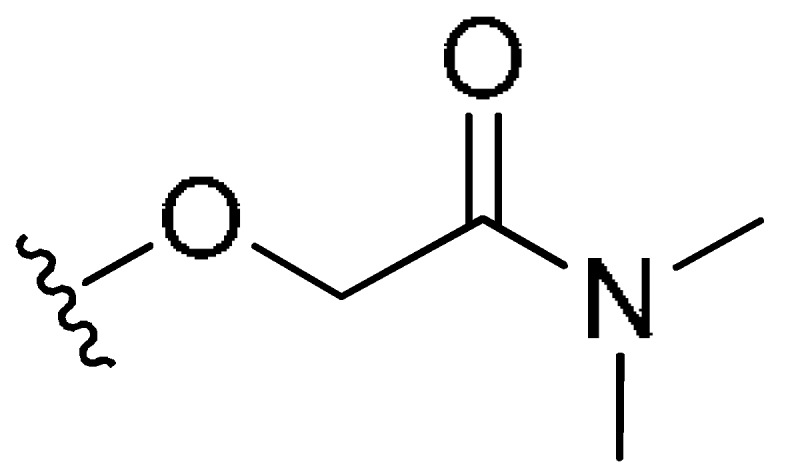	H	0.50	64.57	−2.11
**M32**	CH_3_	CH_3_	Cl	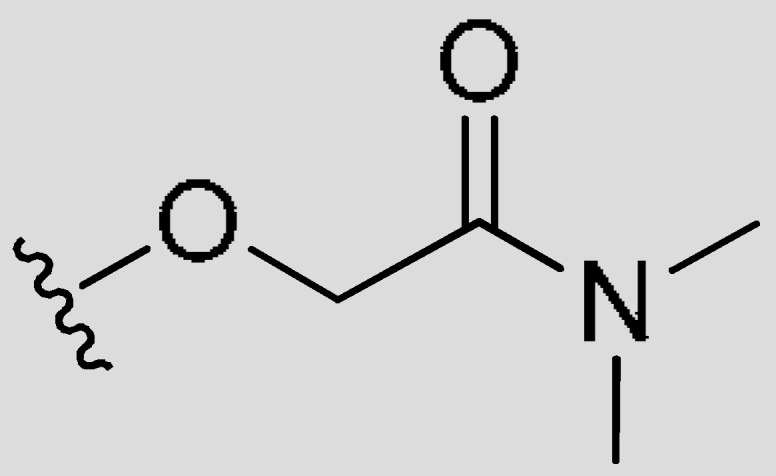	H	81.28	0.60	2.13
**M43(7a)**	H	CH_3_	H	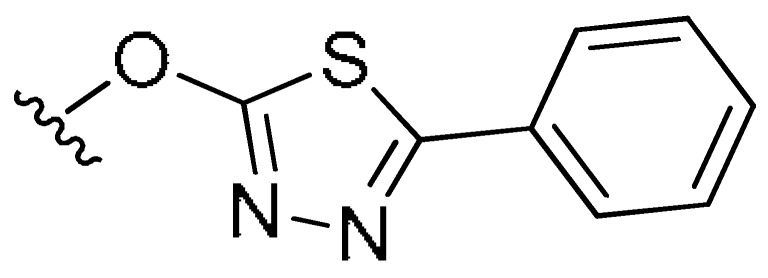	H	0.16	0.36	−0.35
**FR1**	–(CH_2_)_3_–		H	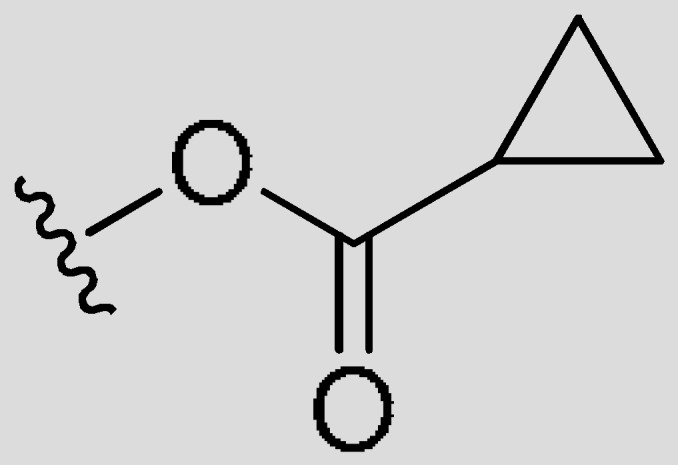	CH_3_	0.0015	75% (>1 uM)	<−2.82
**FR2**	–(CH_2_)_3_–		H	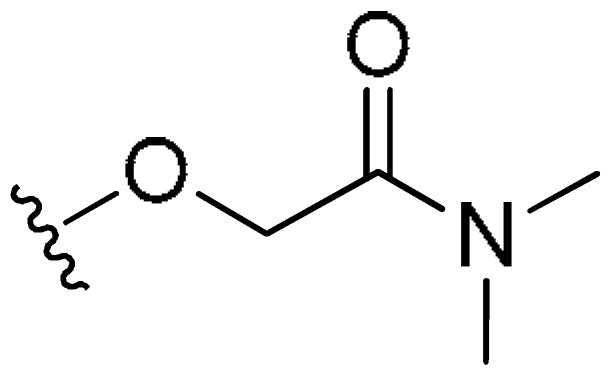	CH_3_	2.82	12% (>10 uM)	<−0.55
**FR3**	–(CH_2_)_4_–		H	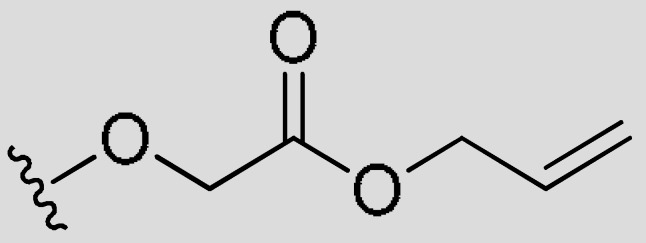	CH_3_	0.74	66% (>5 uM)	<−0.83
**FR4**	–(CH_2_)_3_–		H	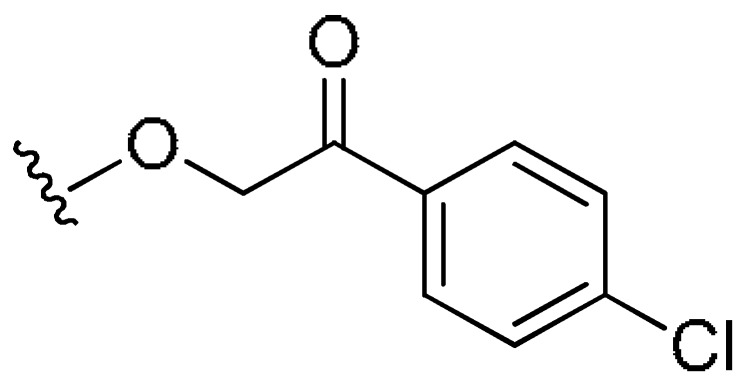	H	23% (>10 uM)	0.018	>2.74
**FR5**	H	CH_3_	H	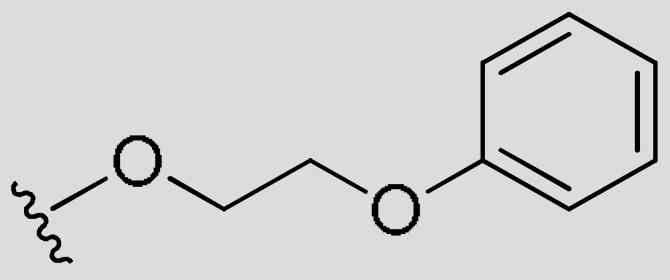	H	28% (>10 uM)	0.015	>2.82
**SP1**	H	CH_3_	H	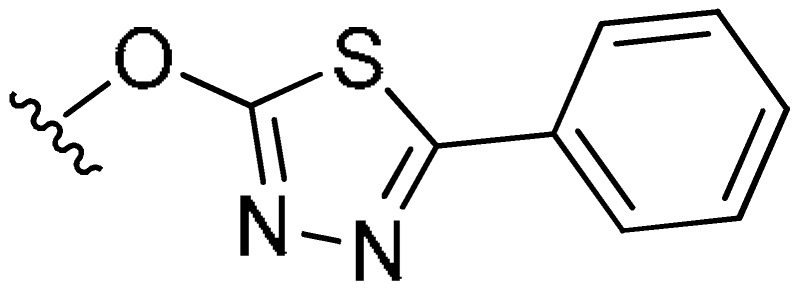	CH_3_	0.019	64% (>5 uM)	<−2.42
**5d**	–(CH_2_)_3_–		H	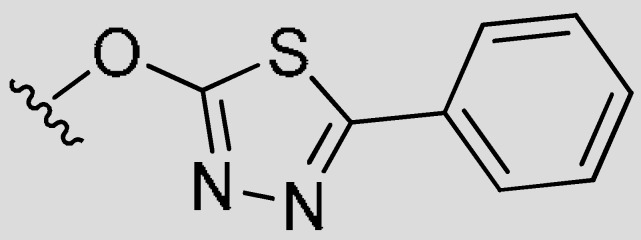	CH_3_	0.096	2.30	−1.38
**7b**	H	(CH_2_)_2_CH_3_	H	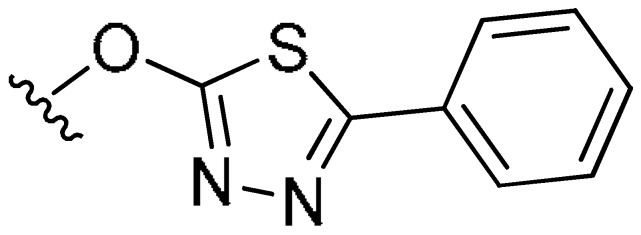	H	0.024	0.01	0.38
**7k**	H	(CH_2_)_2_CH_3_	H	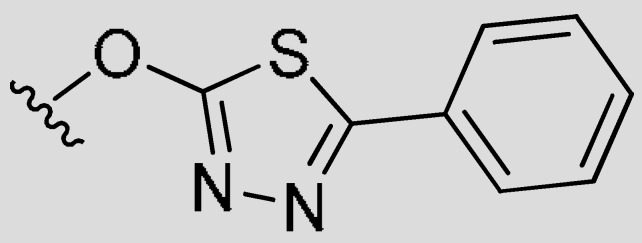	CH_3_	0.021	0.12	−0.76
**7c**	CH_3_	CH_3_	Cl	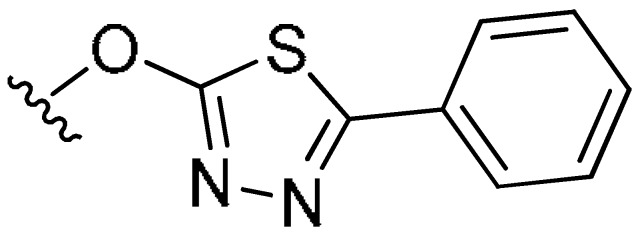	H	0.81	0.44	0.27
**7g**	H	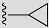	H	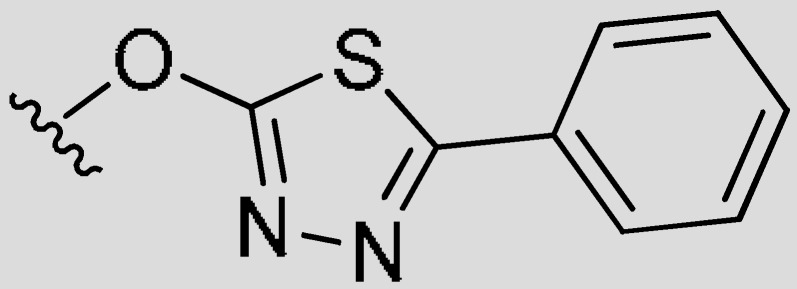	H	2.00	6.20	−0.49

^a^: IC_50_ values (μM) or inhibition activity at 10 μM against *h*MAO-A and *h*MAO-B. ^b^: SI is the selectivity index expressed as pIC_50_(MAO-B)–pIC_50_(MAO-A).

**Table 5:** Calculated physicochemical and pharmacokinetic parameters.

Compounds	logP	MW	Lipinski	Lead-like	Caco-2	CNS
**M29**	1.94	275.3	0 violations	0 violations	Highly permeable	Penetrant
**M31**	2.21	289.33	0 violations	0 violations	Highly permeable	Penetrant
**M32**	2.98	309.74	0 violations	0 violations	Highly permeable	Penetrant
**M43 (7a)**	3.79	336.37	0 violations	0 violations	Highly permeable	Penetrant
**FR1**	3.3	284.31	0 violations	0 violations	Highly permeable	Penetrant
**FR2**	2.32	301.34	0 violations	0 violations	Highly permeable	Penetrant
**FR3**	4.33	328.36	0 violations	1 violation	Highly permeable	Penetrant
**FR4**	4.57	354.78	0 violations	1 violation	Highly permeable	Penetrant
**FR5**	3.77	296.32	0 violations	0 violations	Highly permeable	Penetrant
**SP1**	3.97	350.39	0 violations	0 violations	Highly permeable	Penetrant
**5d**	4.72	376.43	0 violations	1 violation	Highly permeable	Penetrant
**7b**	4.49	364.42	0 violations	1 violation	Highly permeable	Penetrant
**7k**	4.79	378.45	0 violations	1 violation	Highly permeable	Penetrant
**7c**	4.99	384.84	0 violations	1 violation	Highly permeable	Weak Penetrant

## Supplementary Materials

The following are available online at https://www.mdpi.com/1420-3049/24/21/4003/s1, Figure S1: The residue differences of binding pockets between *h*MAO-A and *h*MAO-B. FAD cofactor is shown as space-filling model, MAO-A ligand as green sticks and MAO-B ligand as orange sticks. Figure S2: Predicted binding modes of **M31**, **M32** and **M43** with *h*MAO-A and *h*MAO-B active site. FAD cofactor is shown as space-filling model and compound as green sticks. The H-bond receptor surfaces are shown in pink and the H-bond acceptor surfaces are shown in green. Figure S3: RMSD plots of **M31**, **M32**, **M43** and **C18** with different *h*MAO subtypes. Figure S4: Per-residue RMSF curves of the *h*MAO-A and *h*MAO-B systems. Figure S5: The labeled key motifs of *h*MAO. Loop area is colored red. Alpha helix is colored magenta. Beta sheet is colored orange. Figure S6: RMSD plots for 16 systems. Table S1: Hydrogen bond analysis of four systems according to MD trajectories. Table S2: Free energy decomposition results (kJ/mol) of *h*MAO-A and *h*MAO-B pocket residues. Table S3: pIC_50_ values of synthesized Esuprone derivatives.
