# Supplementary material for: Novel C7-Substituted Coumarins as Selective Monoamine Oxidase Inhibitors: Discovery, Synthesis and Theoretical Simulation
Source: Molecules. 2019 Nov 5;24(21):4003. doi: 10.3390/molecules24214003 (PMC6864482; doi:10.3390/molecules24214003)
Supplement: Supplementary file 1 [file molecules-24-04003-s001.pdf]

Supporting Information

Novel C7-Substituted Coumarins as Selective Monoamine Oxidase Inhibitors: Discovery, Synthesis and Theoretical Simulation

Dong Wang [1,2,†], Ren-Yuan Hong [3,†], Mengyao Guo [2], Yi Liu [2], Nianhang Chen [2], Xun Li [4,*] and De-Xin Kong [1,2,*]

[1] State Key Laboratory of Agricultural Microbiology, Huazhong Agricultural University, Wuhan 430070, China; duke.e.wang@gmail.com (D.W.)

[2] Agricultural Bioinformatics Key Laboratory of Hubei Province, College of Informatics, Huazhong Agricultural University, Wuhan 430070, China; guomengyao96@gmail.com (M.G.); dengxue@webmail.hzau.edu.cn (Y.L.); nhchen95@gmail.com (N.C.)

[3] Key Laboratory of Chemical Biology (Ministry of Education), School of Pharmaceutical Sciences, Shandong University, 44 West Culture Road, 250012, Ji'nan, Shandong, China; 17865132563@163.com (R.H.)

[4] Institute of Materia Medica, Shandong First Medical University & Shandong Academy of Medical Sciences, No 18877, Jingshi Road, 250002, Ji'nan, Shandong, China;

* Correspondence: tjulx2004@sdu.edu.cn (X.L.); dxkong@mail.hzau.edu.cn (D.X.K.); Tel.: 86-531-88382005 (X.L.); +86-27-8728 0877 (D.X.K.)

† The first two authors contributed equally.

1.1 *Molecular docking studies.*

Enlightened by the above-mentioned SARs, three compounds, **M31**, **M32** and **M43**, were selected for docking simulations, aiming to provide a relatively reliable initial conformation in *h*MAO-A and *h*MAO-B for the next MD simulations. As illustrated in Figure S1, there were 7 different amino acids of the 24 residues in the two MAO subtypes. Among the 7 amino acids, it was worth noting that residue Ile199 acted as a gatekeeper between the entrance cavity and the substrate cavity with different conformations. Figure S2 showed the predicted binding poses of the 3 compounds in both *h*MAO-A and *h*MAO-B (PDB ID: 2Z5X and 2V61, respectively). As shown in Figure S2., these three coumarin derivatives which has different C7 substitutions bind to the active site with coumarin moiety ring towards the substrate cavity. It can be observed that these three C7 substituted coumarins all formed interactions with important residues, such as Phe208 and Gln215 in *h*MAO-A, Ile199 and Cys172 in *h*MAO-B. An extended ligand conformation of these 3 compounds was found after docking in *h*MAO-B. More specifically, the hydrogen bond between compound **M32** and Ile199 could keep the gate open. However in *h*MAO-A, Ile199 residue was replaced by Phe208, which forced the ligand to adopt a more curved conformation. Starting from the optimal docked models, eight different simulation systems were therefore investigated to elucidate the selectivity mechanism, in which **C18** was the co-crystallized ligand in 2V61 (MAO-A *v.s.* **M31**, MAO-A *v.s.* **M32**, MAO-A *v.s.* **M43**, MAO-A *v.s.* **C18**, MAO-B *v.s.* **M31**, MAO-B *v.s.* **M32**, MAO-B *v.s.* **M43**, MAO-B *v.s.* **C18**).

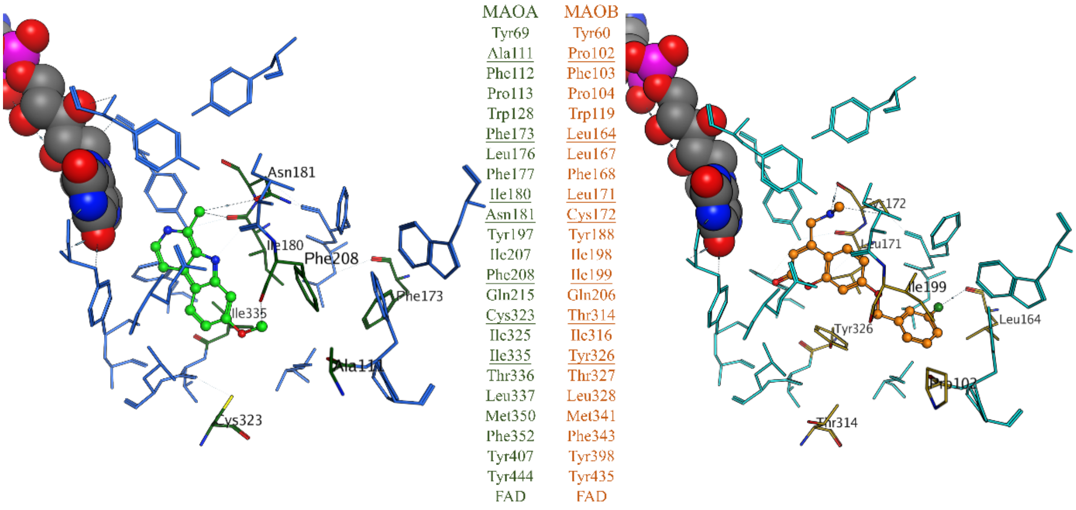

Figure S1. The residue differences of binding pockets between *h*MAO-A and *h*MAO-B. FAD cofactor is shown as space-filling model, MAO-A ligand as green sticks and MAO-B ligand as orange sticks.

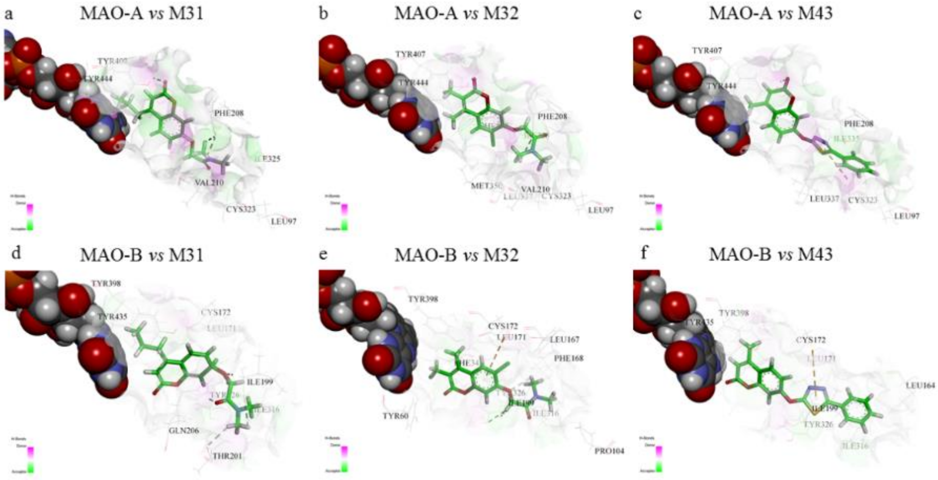

Figure S2. Predicted binding modes of **M31**, **M32** and **M43** with *h*MAO-A and *h*MAO-B active site. FAD cofactor is shown as space-filling model and compound as green sticks. The H-bond receptor surfaces are shown in pink and the H-bond acceptor surfaces are shown in green.

1.2 Evaluation of the structural stability.

In MD simulations, RMSD analysis of the protein backbone was usually regarded as the first step for checking the systems stability. As depicted in Figure S3, the RMSD plots of the eight simulation systems were recorded with respect to the initial conformation at 0 ns over the 20 ns MD simulation. As clearly shown in the plots, only slight RMSD fluctuations were observed after 10 ns of simulation. This indicated that all the simulation systems achieved a relatively stable conformation during the equilibration period. Larger fluctuations were

observed more frequently in MAO-A systems than in MAO-B systems and this may due to the low resolution in the X-ray structure of the former protein. For each MAO subtypes, the complexes with the selective inhibitor have fewer fluctuations compared to the complexes with non-selective ligands. The stability of the selective complex systems was validated by the hydrogen bonds analysis between MAO subtypes and C7 substituted coumarins.

The regional conformational fluctuations of each residue were assessed by calculating the root-mean square fluctuation (RMSF). Similarly, RMSF profiles of main chains for eight simulation systems were depicted in Figure S4. In order to illustrate the different fluctuation trends of key regions in the binding site, three essential parts, the loop region, the α-helix and β-sheet were labeled in different colors in Figure S5. The residues with comparatively larger fluctuation suggested that they could adopt more flexible conformations during the whole MD simulations. As shown in Figure S4, the α–helix and the β-sheet regions of two subtypes exhibited small fluctuation patterns, indicating these two regions were more stable than loop regions. The decreased flexibility in the α-helix and the β-sheet region can be explained by stabilizing protein-ligand interactions between selective inhibitors and some key residues of MAOs (**M31** *v.s.* MAO-A, **M32** *v.s.* MAO-B). Moreover, **M31** *v.s.* MAO-A system had more fluctuations than **M43** systems in loop regions, since the C7 substituent of **M31** has higher number of rotatable bonds which cause the destabilization of the system. It could also be observed that selective systems had fewer larger fluctuations, which was consistent with the result of RMSD analysis.

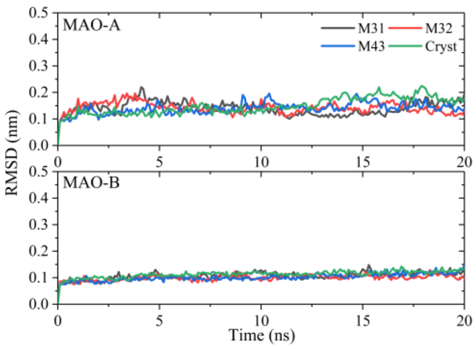

Figure S3. RMSD plots of **M31**, **M32**, **M43** and **C18** with different *h*MAO subtypes.

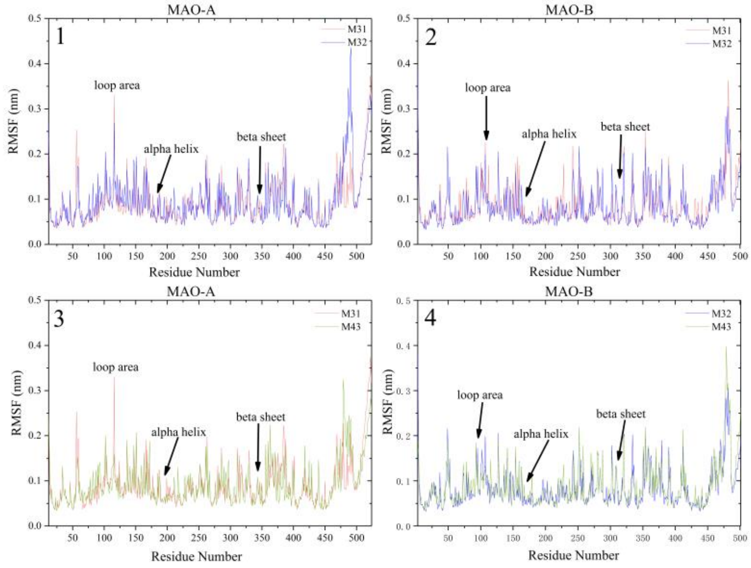

Figure S4. Per-residue RMSF curves of the *h*MAO-A and *h*MAO-B systems.

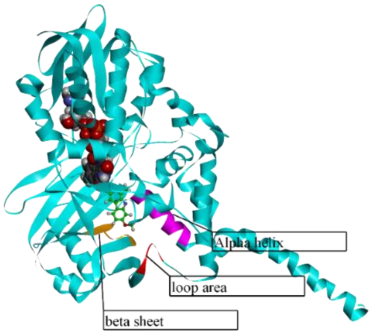

Figure S5. The labeled key motifs of *h*MAO. Loop area is colored red. Alpha helix is colored magenta. Beta sheet is colored orange.

1.3 Hydrogen bonds Analysis.

The stability of hydrogen bonds interactions between *h*MAO and C7 substituted coumarins which are likely to contribute to the selectivity of MAOIs was investigated through the MD simulations. VMD 1.9.2 was used to analyze the hydrogen bonds in each of the eight simulation systems. The distance cutoff value was defined as 3.5 Å and the angle cutoff value was set as 35°. Only the occupancy over 5% during the equilibrium period was considered for analysis and the detailed results were listed in Table S1. Dual inhibitor **M43** formed H-bonds with residues in the α-helix region of two MAO subtypes, such as Asn181 of *h*MAO-A and Cys172 of *h*MAO-B. **M31** which is a selective inhibitor of MAO-A forms a dominant hydrogen bond interaction with residue Gln215 of α-helix region (occupancy = 95%), which may play a key role in *h*MAO-A selectivity. As for **M32** or **C18** against *h*MAO-B selective systems, H-bonds between Cys172 and Ile199 with low occupancy are observed. It was interesting that hydrogen

bonds formed by Tyr residues (Tyr326, Tyr398 and Tyr435), which caused the formation of a hydrophobic cage in *h*MAO-B binding site were stable during the MD simulations. These important hydrogen bond interactions stabilize a binding cavity and facilitating the binding of the inhibitors in *h*MAO-B receptor. The analysis suggested that a constant interaction with Gln215 from the α-helix would enhance the selectivity against *h*MAO-A. This observation is in agreement with the results of Giuseppe et al.[1]. Hydrogen bond between Cys172 and the inhibitor also contributed to the MAO-B selectivity since Asn181 in *h*MAO-A took the place of Cys172 in *h*MAO-B. Based on hydrogen bond analysis, a rational way to enhance MAO-subtype selectivity can be proposed. For instance, *h*MAO-A selectivity would be improved if the hydrogen bond interaction strength with Gln215 was enhanced. Structural modification to form a stable interaction with the hydrophobic cage could strengthen the activity against *h*MAO-B.

Table S1. Hydrogen bond analysis of four systems according to MD trajectories.

Systems	Donor	Acceptor	Occupancy
**M31** *v.s.* MAO-A	Gln215-Side	**M31**	95.1%
	Asn181-Side	**M31**	23.3%
	Ile207-Side	**M31**	10.4%
	Phe208-Side	**M31**	9.4%
**M31** *v.s.* MAO-B	Tyr326-Side	**M31**	80.7%
	Phe343-Side	**M31**	9.4%
**M32** *v.s.*MAO-A	Tyr197-Side	**M32**	77.7%
	Ile207-Side	**M32**	8.4%
	Gln215-Side	**M32**	53.5%
	**M32**	Asn181-Side	21.8%
**M32** *v.s.* MAO-B	Tyr326-Side	**M32**	59.4%
	**M32**	Cys172-Main	5.5%
	**M32**	Ile199-Main	8.4%
**M43** *v.s.*MAO-A	Tyr197-Side	**M43**	72.8%
	Asn181-Side	**M43**	11.4%
	Asn181-Main	**M43**	18.3%
	**M43**	Asn181-Side	11.4%
**M43** *v.s.* MAO-B	**M43**	Cys172-Main	5.5%
	**M43**	Tyr435-Side	8.9%
**C18** *v.s.* MAO-A	Phe208-Main	**C18**	40.1%
	**C18**	Asn181-Side	62.4%
	**C18**	Asn181-Main	51.0%
	**C18**	Tyr444-Side	26.7%
	**C18**	Thr336-Main	14.9%
	Ile207-Side	**C18**	19.3%
	**C18**	Phe208-Main	10.4%
	**C18**	Tyr407-Side	8.4%
**C18** *v.s.* MAO-B	**C18**	Cys172-Main	21.8%
	**C18**	Tyr435-Side	22.8%

	**C18**	Tyr398-Side	20.3%
	Ile199-Main	**C18**	5.5%

*Compound **M29**-**M34** and **M43** were obtained based on our BRS-3D VS protocol. **C18** was the self-contained ligand in 2V61.

Table S2. Free energy decomposition results (kJ/mol) of *h*MAO-A and *h*MAO-B pocket residues.

**Residues**		**MAO-A *v.s.* M43**	**MAO-B *v.s.* M43**	**MAO-A *v.s.* C18**	**MAO-B *v.s.* C18**
**Tyr69**	Tyr60	-2.1699 +/- 0.0485	-1.4899 +/- 0.0555	-2.2074 +/- 0.0487	-3.2926 +/- 0.0682
**Ala111**	Pro102	1.2416 +/- 0.1103	2.2244 +/- 0.1224	1.0437 +/- 0.1108	2.4398 +/- 0.1487
**Phe112**	Phe103	-0.7385 +/- 0.0290	0.3891 +/- 0.0531	1.0733 +/- 0.0554	0.8511 +/- 0.0559
**Pro113**	Pro104	-0.1703 +/- 0.0073	-1.9962 +/- 0.1090	-0.1157 +/- 0.0595	-0.6065 +/- 0.0435
**Trp128**	Trp119	0.0561 +/- 0.0050	-1.1615 +/- 0.0762	-0.1526 +/- 0.0090	-0.3703 +/- 0.0289
**Phe173**	Leu164	-0.0873 +/- 0.0097	-1.9611 +/- 0.1391	-0.5923 +/- 0.0153	-1.8558 +/- 0.0540
**Leu176**	Leu167	-0.0459 +/- 0.0127	-2.4919 +/- 0.0868	-0.8342 +/- 0.0254	-1.4404 +/- 0.0606
**Phe177**	Phe168	0.3149 +/- 0.0327	-6.0771 +/- 0.1701	-2.8121 +/- 0.0751	-3.4035 +/- 0.0838
**Ile180**	Leu171	-6.2327 +/- 0.0959	-6.1374 +/- 0.2283	-4.7420 +/- 0.1668	-9.9879 +/- 0.1790
**Asn181**	Cys172	-1.7920 +/- 0.2895	-4.1876 +/- 0.1293	-19.2334 +/- 0.3718	-8.6903 +/- 0.2828
**Tyr197**	Tyr188	-1.9959 +/- 0.2075	0.8642 +/- 0.0992	-2.1768 +/- 0.0831	-0.8742 +/- 0.1858
**Ile207**	Ile198	-0.5426 +/- 0.1839	-1.7415 +/- 0.1249	-5.9627 +/- 0.1519	-3.5065 +/- 0.1354
**Phe208**	Ile199	-3.4572 +/- 0.2106	-6.8623 +/- 0.2022	-6.7249 +/- 0.3277	-8.1047 +/- 0.2021
**Gln215**	Gln206	-3.6756 +/- 0.1794	-2.5042 +/- 0.1704	-6.1142 +/- 0.2023	-6.0440 +/- 0.2239
**Cys323**	Thr314	-0.8257 +/- 0.0808	-0.7217 +/- 0.0387	-0.2966 +/- 0.0893	-0.1698 +/- 0.0357
**Ile325**	Ile316	-0.8940 +/- 0.0330	-6.0684 +/- 0.1105	-2.0462 +/- 0.1280	-4.1768 +/- 0.1016
**Ile335**	Tyr326	-6.8937 +/- 0.1179	-5.5909 +/- 0.1882	-7.1344 +/- 0.1613	-2.2518 +/- 0.1711
**Thr336**	Thr327	0.0826 +/- 0.0628	0.0767 +/- 0.0205	1.1541 +/- 0.1967	-0.6694 +/- 0.0298
**Leu337**	Leu328	-3.8445 +/- 0.0960	-0.6418 +/- 0.0266	-3.4453 +/- 0.1016	-0.7503 +/- 0.0235
**Met350**	Met341	-0.3784 +/- 0.0178	-0.1216 +/- 0.0091	-0.4051 +/- 0.0440	-0.1350 +/- 0.0266
**Phe352**	Phe343	-0.9866 +/- 0.0386	-1.5778 +/- 0.0633	-0.5815 +/- 0.0494	-1.1044 +/- 0.0894
**Tyr407**	Tyr398	-10.2894 +/- 0.1631	-6.7161 +/- 0.1788	-4.8024 +/- 0.2837	-8.0050 +/- 0.3251
**Tyr444**	Tyr435	-3.9190 +/- 0.2187	-1.2072 +/- 0.1828	-5.3449 +/- 0.1905	-5.7731 +/- 0.2653

Table S3. pIC_{50} values of synthesized Esuprone derivatives.

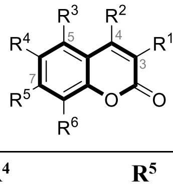

**Compds**	**R^1**	**R^2**	**R^3**	**R^4**	**R^5**	**R^6**	**MAO-A***	**MAO-B***
**5a**	–$(CH_2)_3$–		H	H	$C_2H_5SO_2$	CH_3	0.011	2.3
**5b**	–$(CH_2)_3$–		H	H	*p*-$CH_3C_6H_4SO_2$	CH_3	0.012	4.9
**5c**	–$(CH_2)_3$–		H	H	*p*-$NO_2C_6H_4SO_2$	CH_3	< 0.005	0.59
**7d**	H	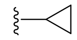	H	H	$C_2H_5SO_2$	H	1.4	> 100
**7e**	H	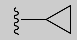	H	H	*p*-$CH_3C_6H_4SO_2$	H	1.1	9.5
**7f**	H	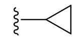	H	H	*p*-$NO_2C_6H_4SO_2$	H	0.095	9
**7h**	H	$(CH_2)_2CH_3$	H	H	$C_2H_5SO_2$	CH_3	0.91	9.9

**7i**	H	$(CH_2)_2CH_3$	H	H	*p*-$CH_3C_6H_4SO_2$	CH_3	0.17	4.1
**7j**	H	$(CH_2)_2CH_3$	H	H	*p*-$NO_2C_6H_4SO_2$	CH_3	0.029	1.7

*: The unit of IC_{50} values is uM. IC_{50} value of Esuprone is equal to 10.7 nM. For Esuprone analogs, four compounds **5a-c** and **7j** had comparable activity with Esuprone. Compound **5c** was the most active MAO-A inhibitor with an IC_{50} value of ~5 nM. Comparing with **7d** and Esuprone, installation of cyclopropyl group at C4-position significantly decreased the MAO-A inhibition. However, flexible substitutes at C4-position can be accommodated for MAO-A inhibition (**7h-j**), which indicated large rigid substitutions were not suitable for MAO-A inhibitors It can also be observed that changing the substituent at C7 position generally influenced the inhibition activity towards MAO-A (**5a-c**).

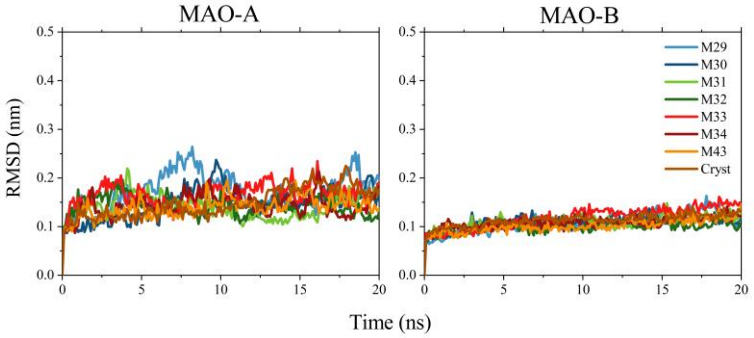

Figure. S6. RMSD plots for 16 systems.

References

1. Mangiatordi, G.F.; Alberga, D.; Pisani, L.; Gadaleta, D.; Trisciuzzi, D.; Farina, R.; Carotti, A.; Lattanzi, G.; Catto, M.; Nicolotti, O. A rational approach to elucidate human monoamine oxidase molecular selectivity. *Eur J Pharm Sci* **2017**, *101*, 90-99, doi:10.1016/j.ejps.2017.02.008.

The ^{1}H NMR spectra of Compound 1

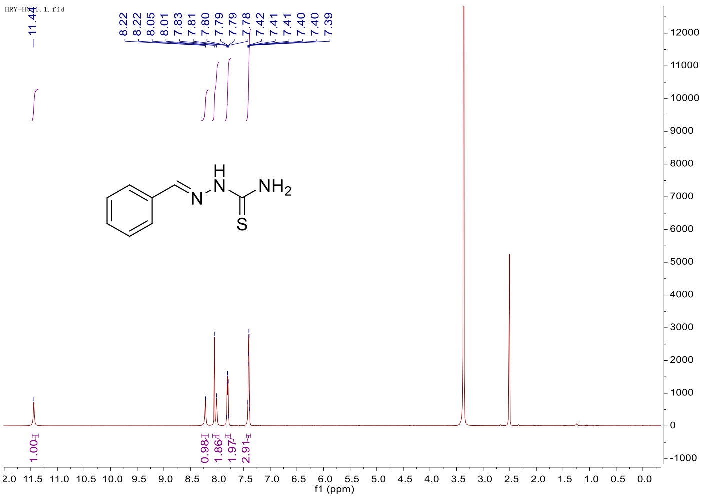

The ^{1}H NMR spectra of Compound 2

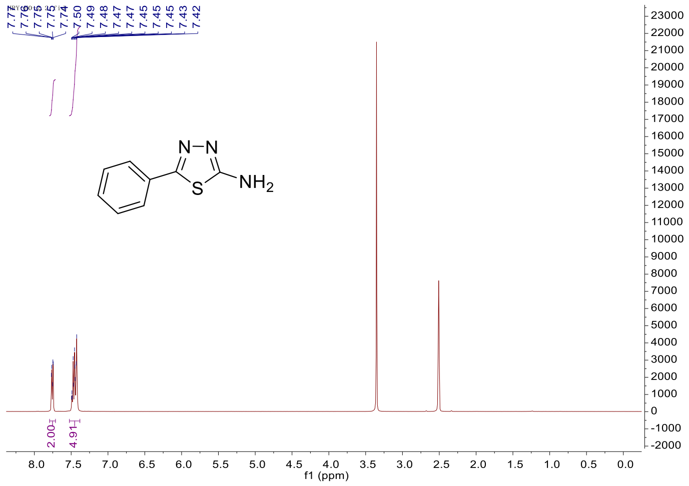

The ^{1}H NMR, ^{13}C NMR spectra of Compound 3

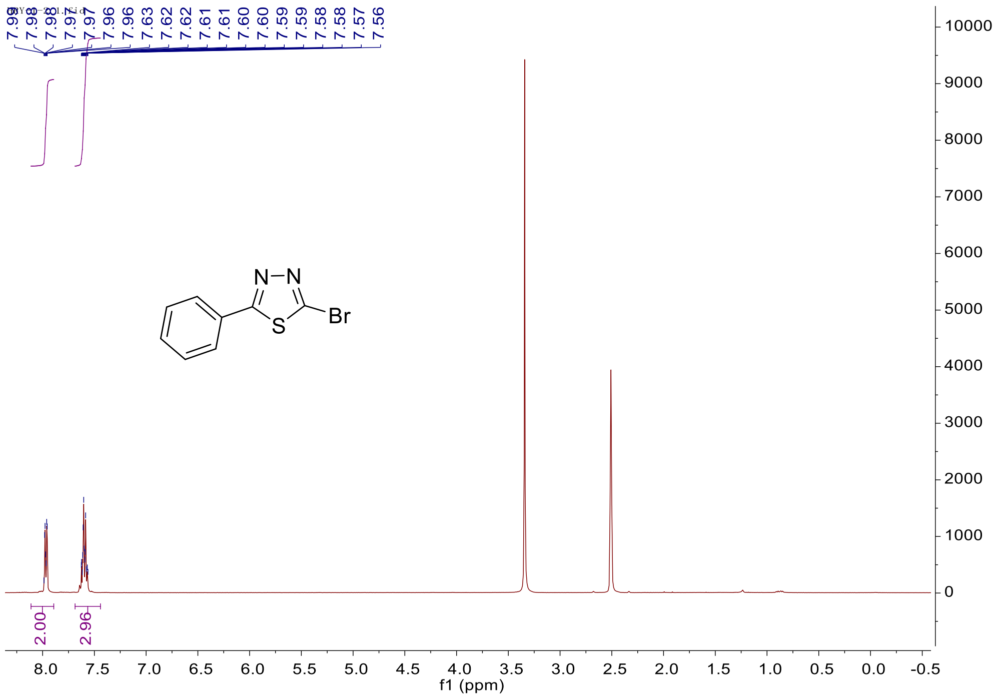

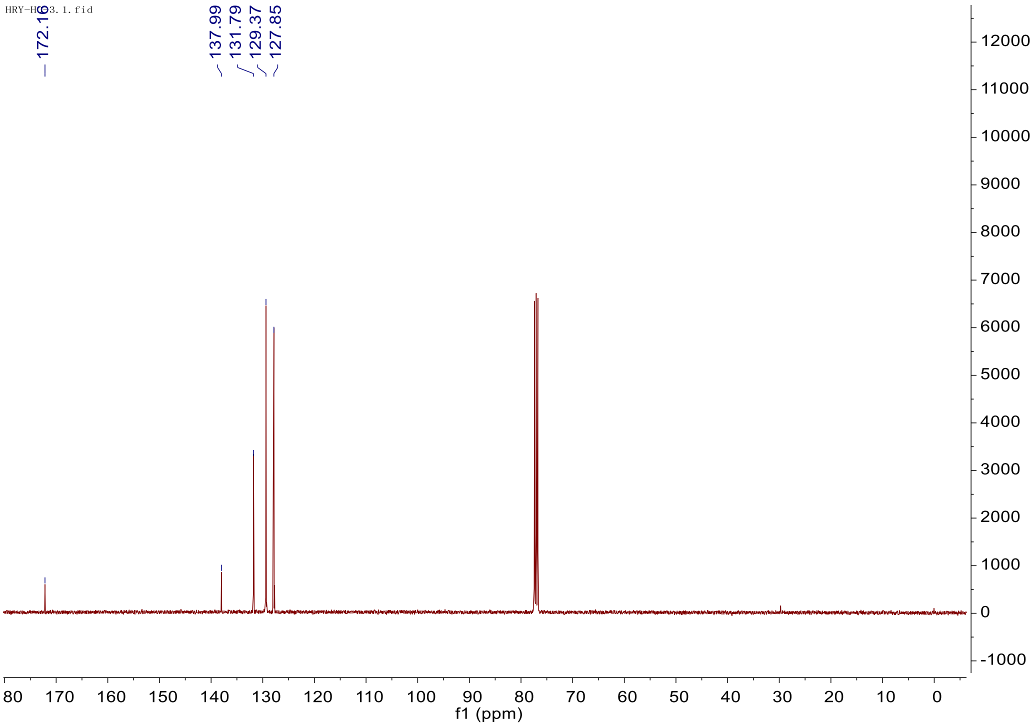

The ¹H NMR spectra of Compound 4

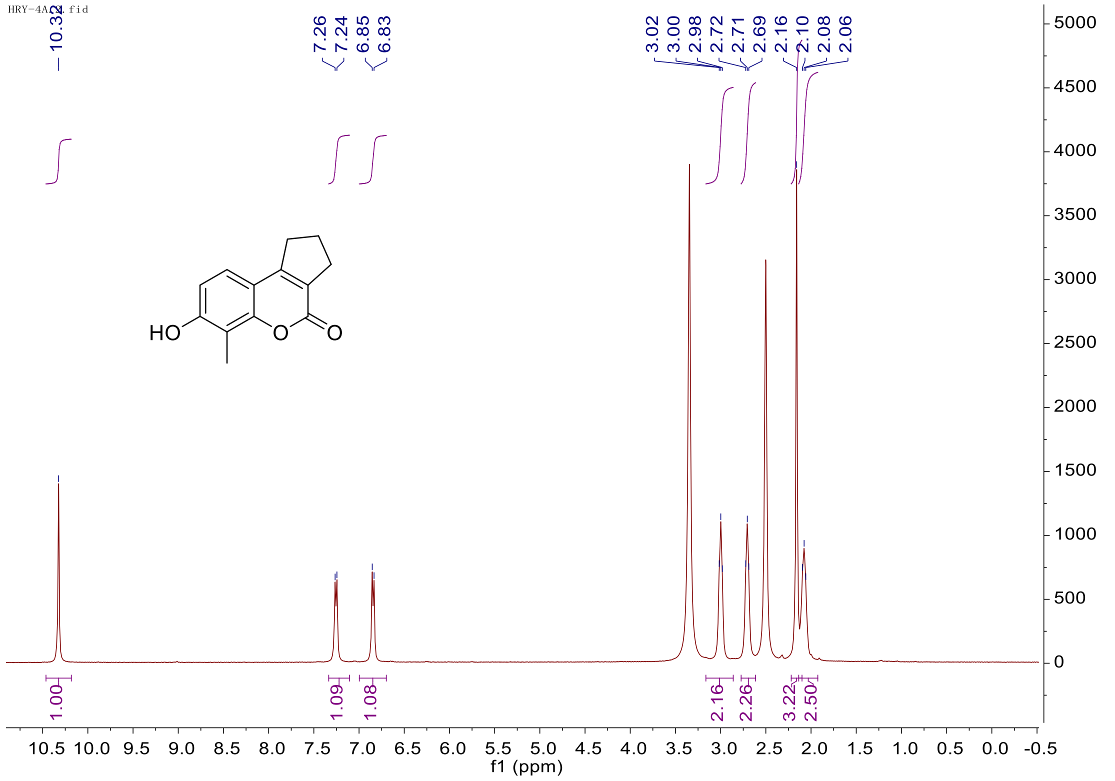

The ¹H NMR spectra of Compound 6a

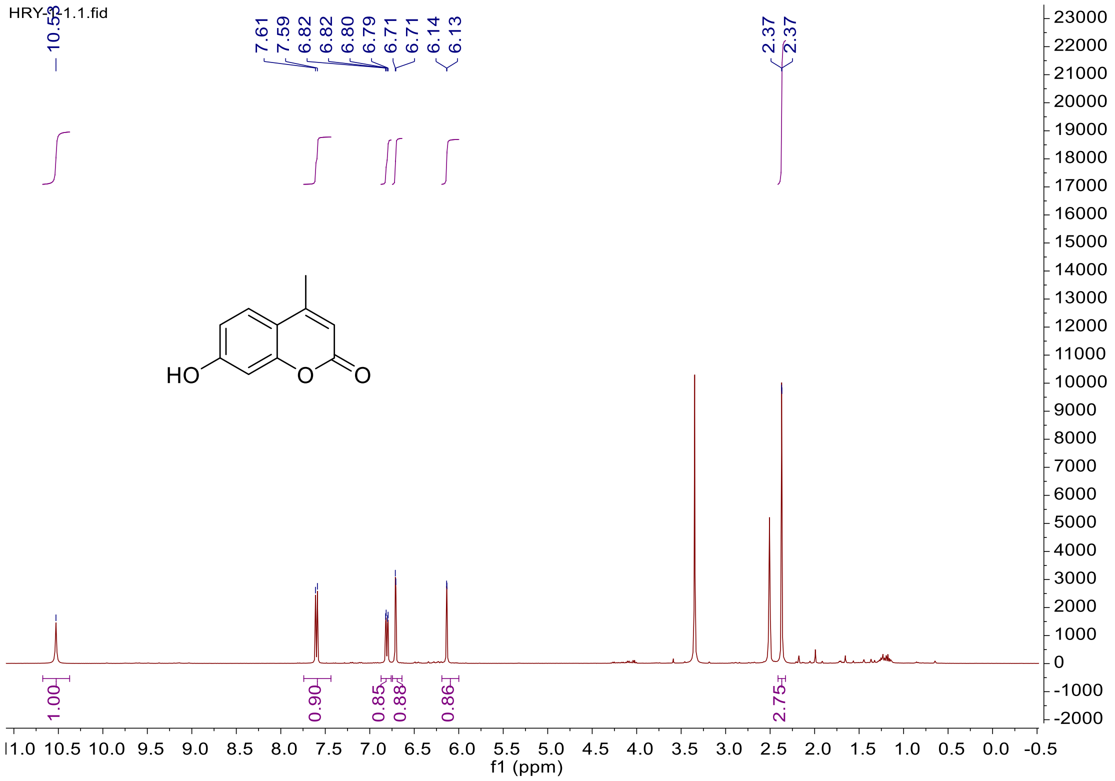

The ^{1}H NMR spectra of Compound 6b

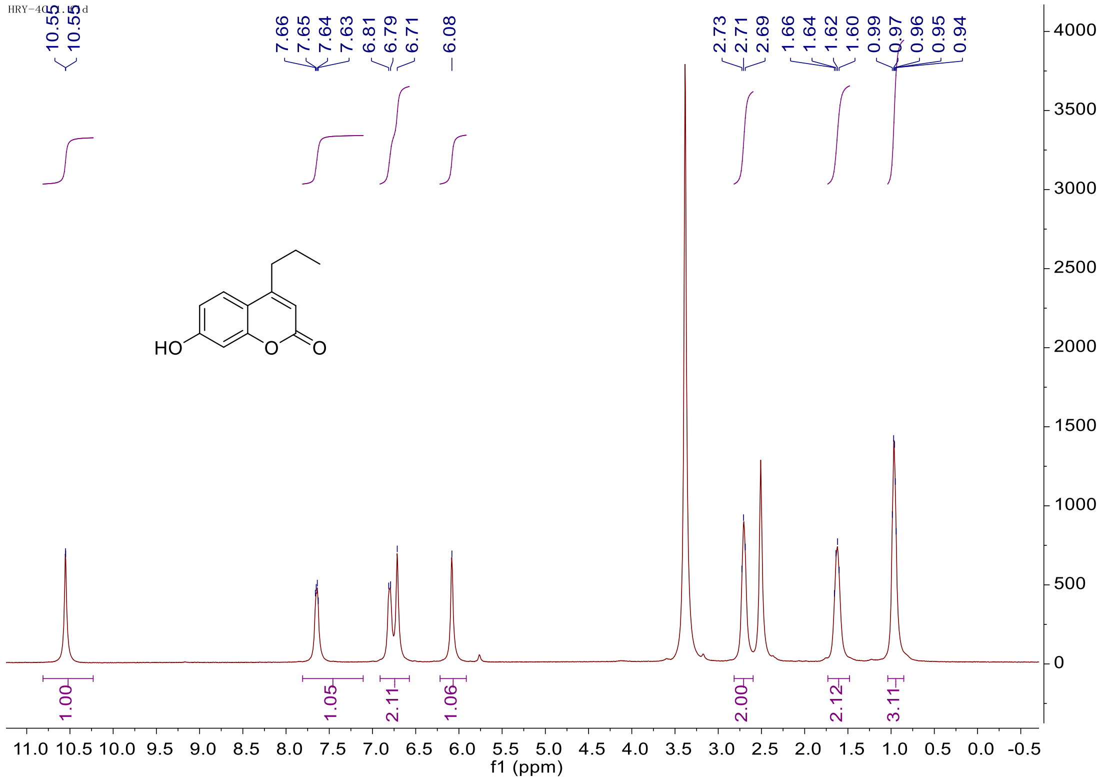

The ^{1}H NMR spectra of Compound 6c

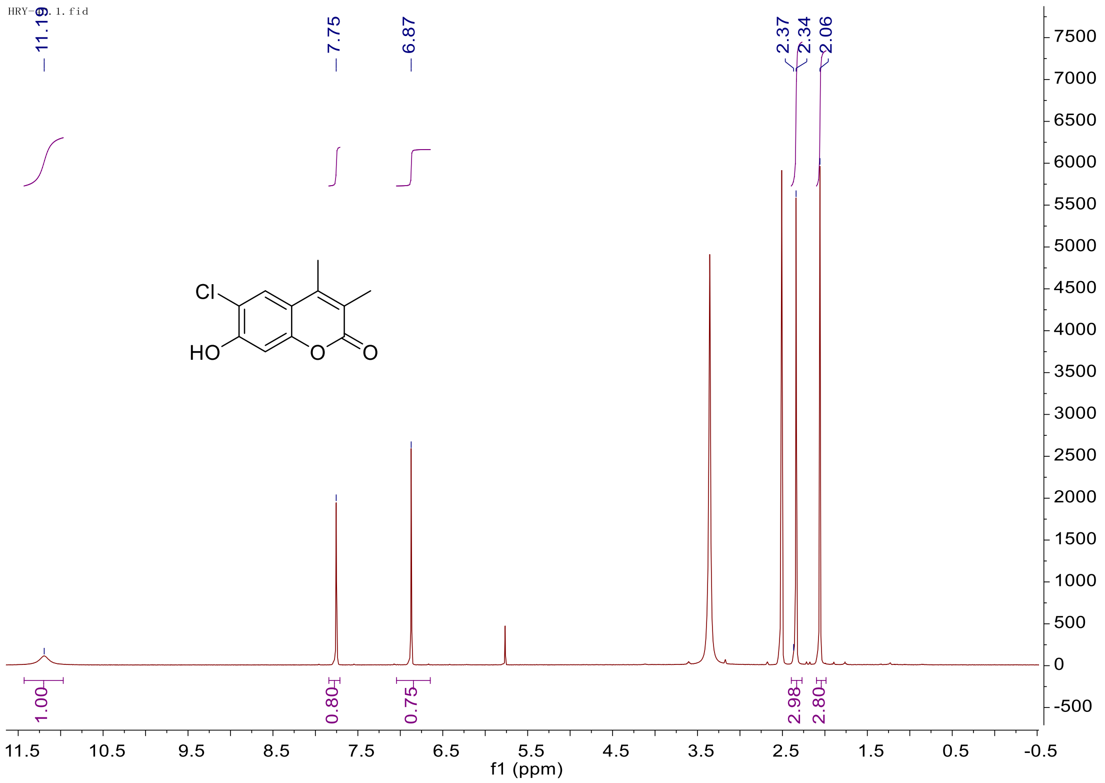

The ^{1}H NMR spectra of Compound 6d

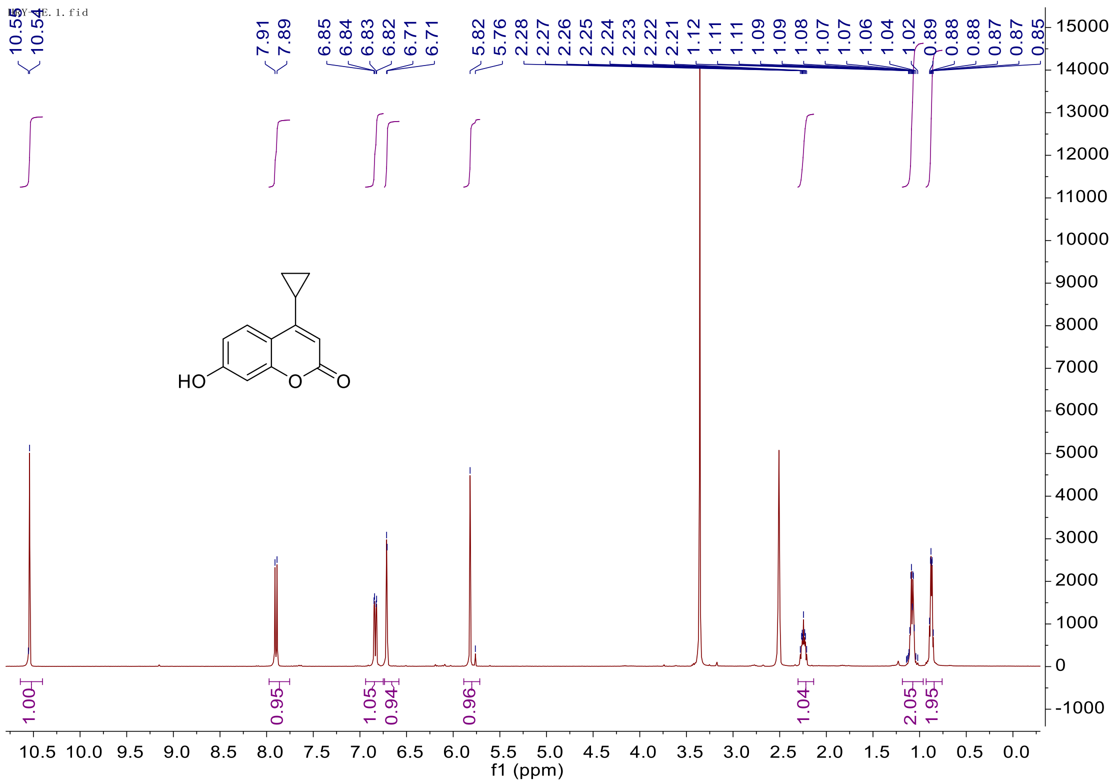

The ^{1}H NMR spectra of Compound 6e

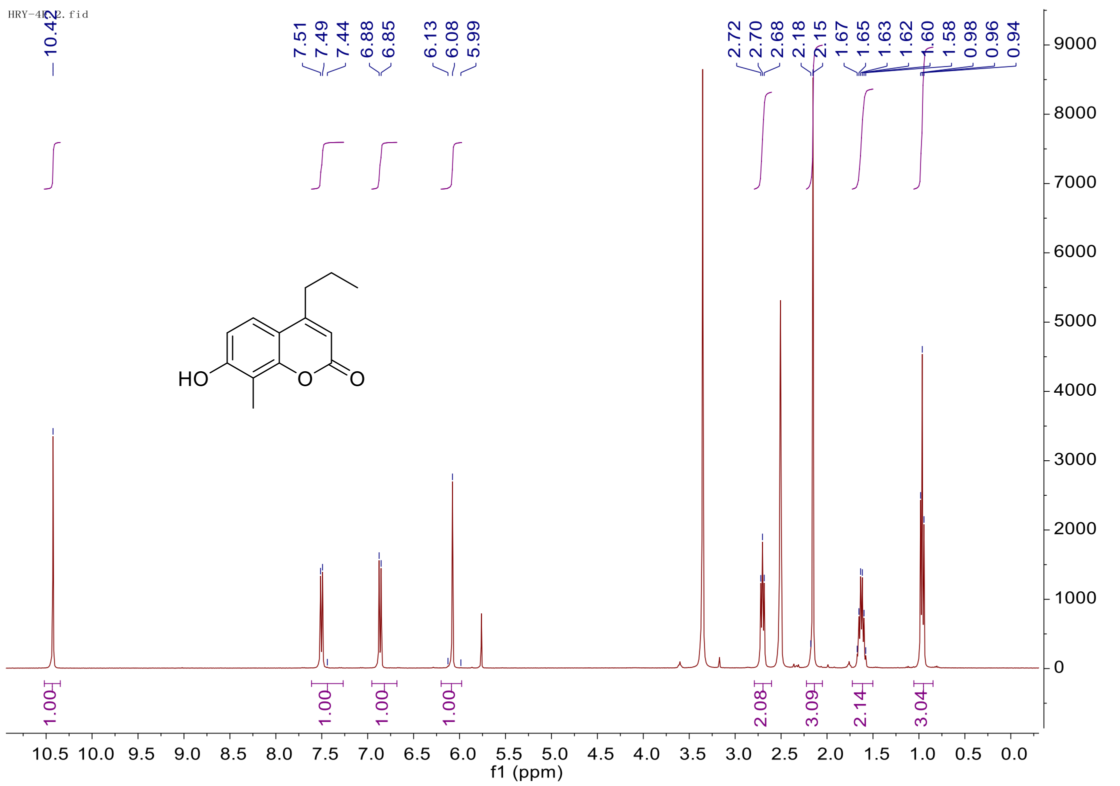

The ^{1}H NMR, ^{13}C NMR spectra of Compound 5d

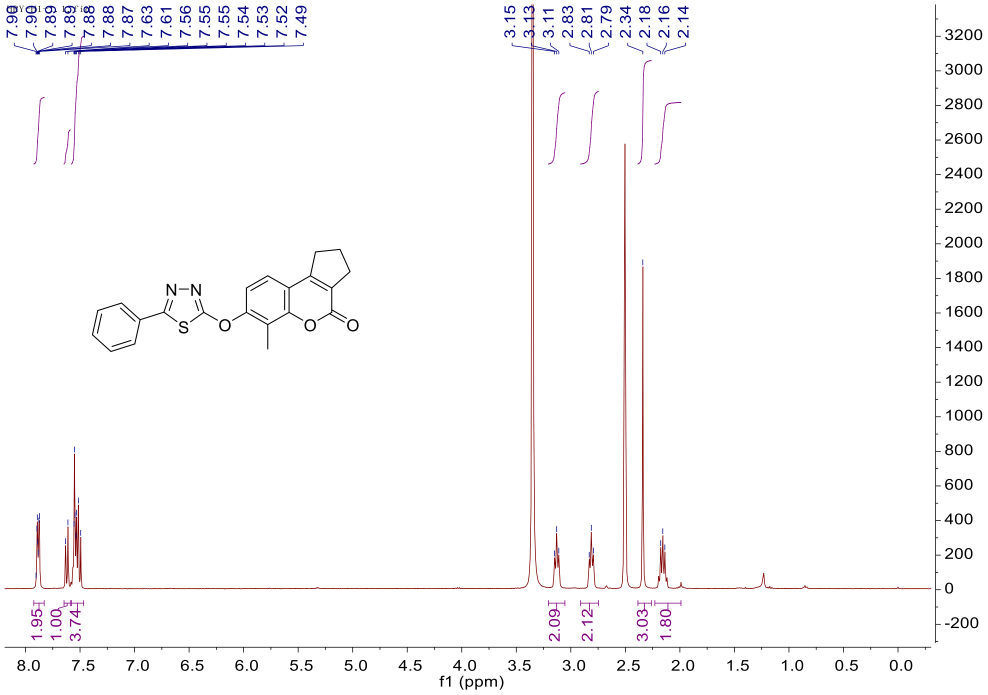

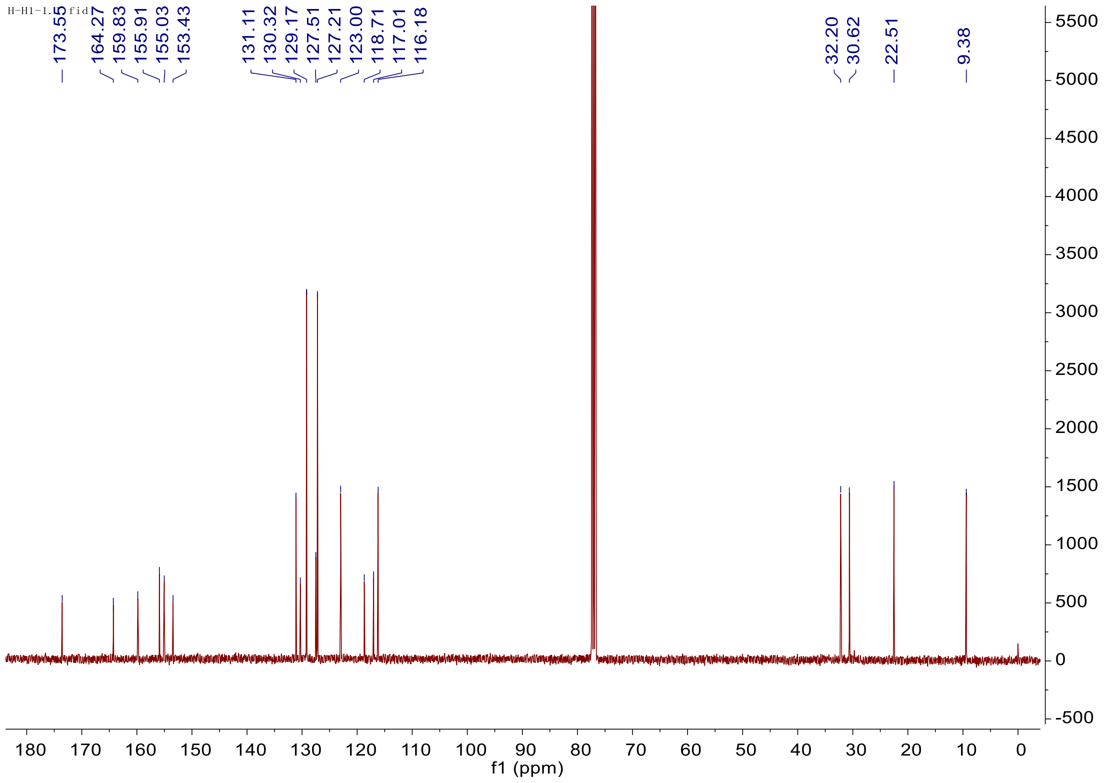

The ^{1}H NMR, ^{13}C NMR spectra of Compound 7a

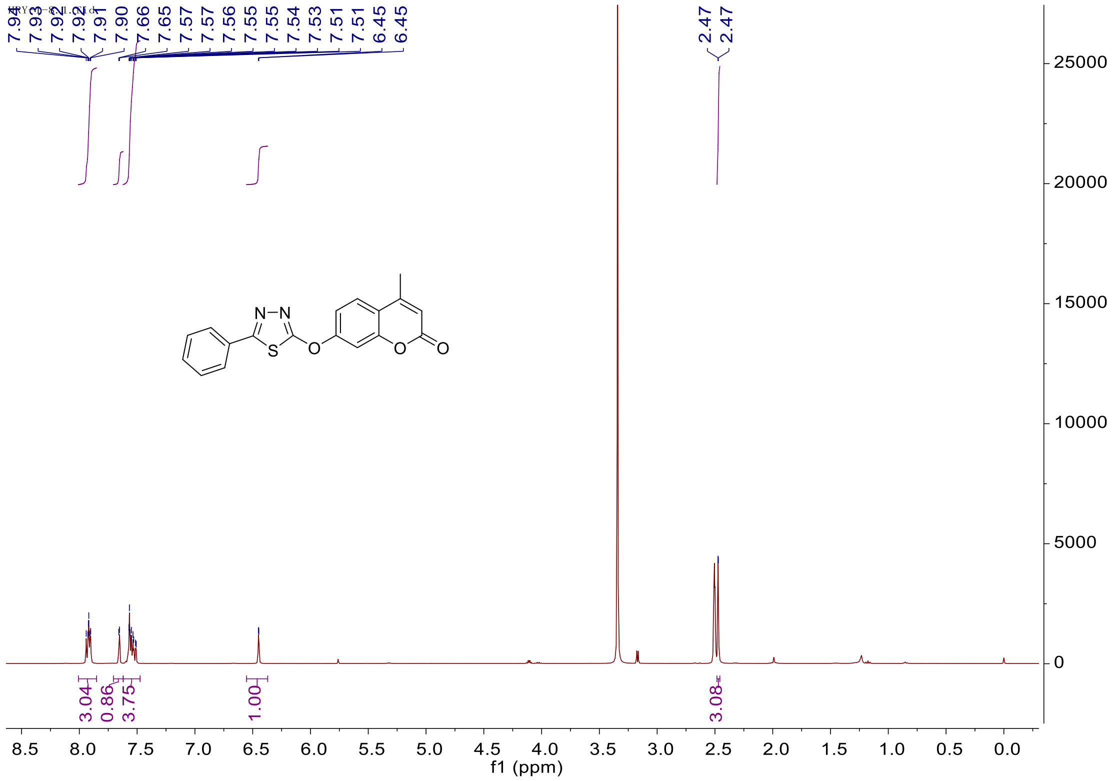

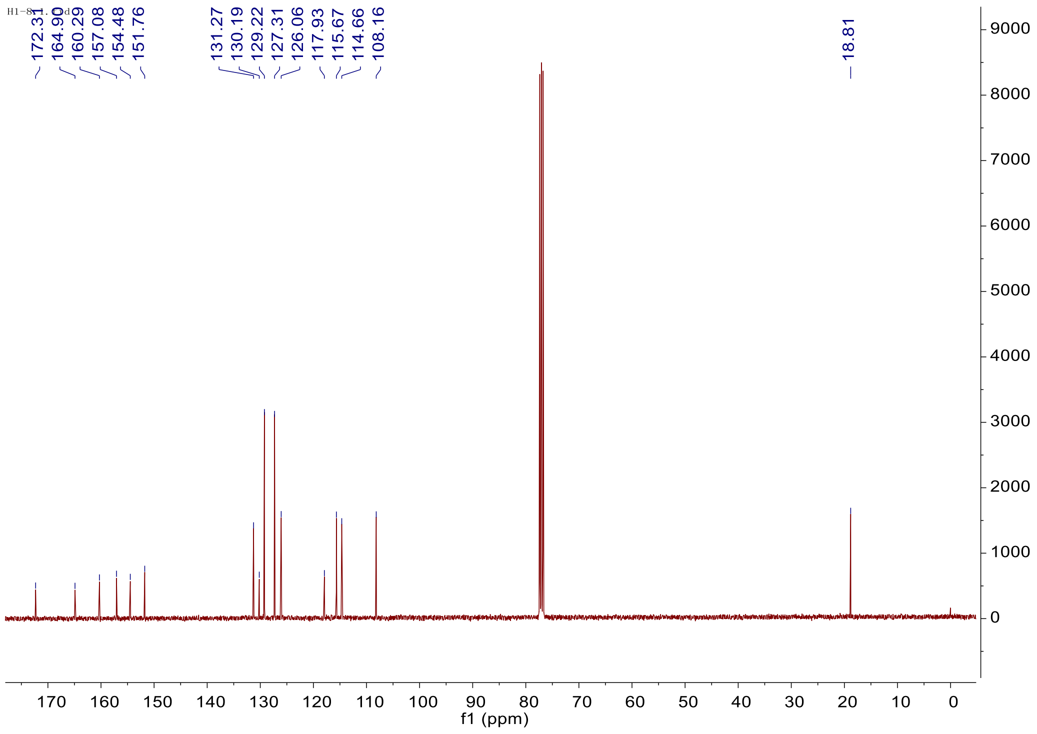

The ^{1}H NMR, ^{13}C NMR spectra of Compound 7b

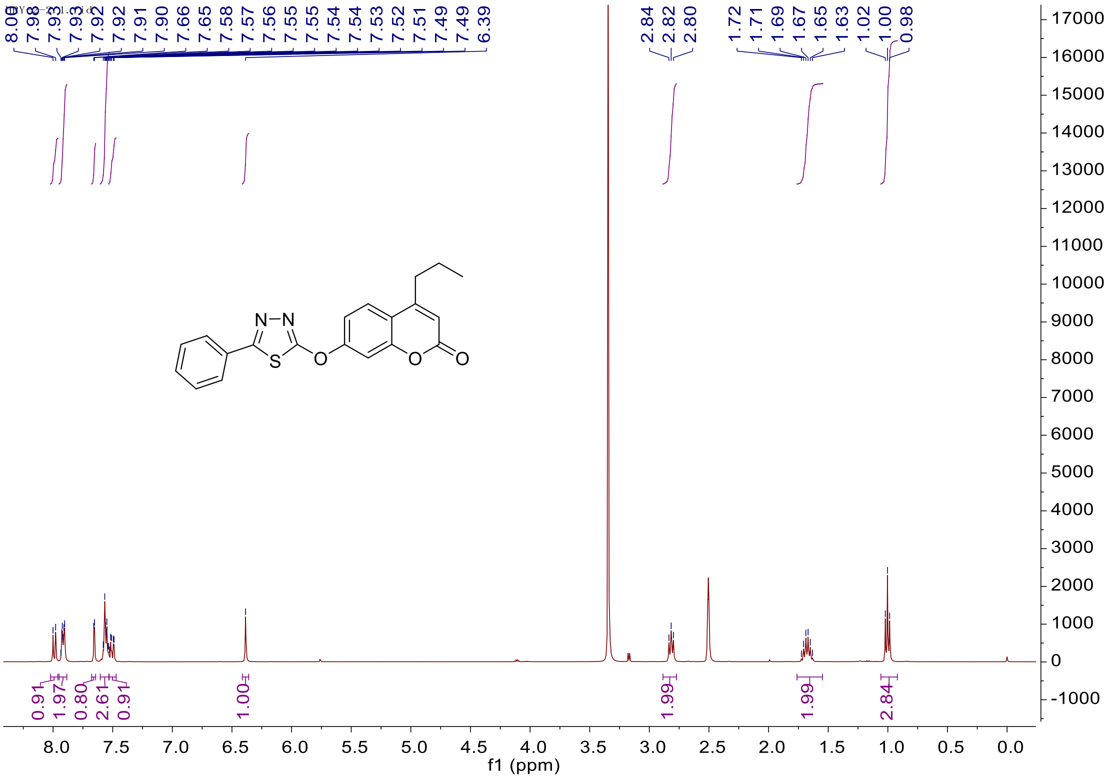

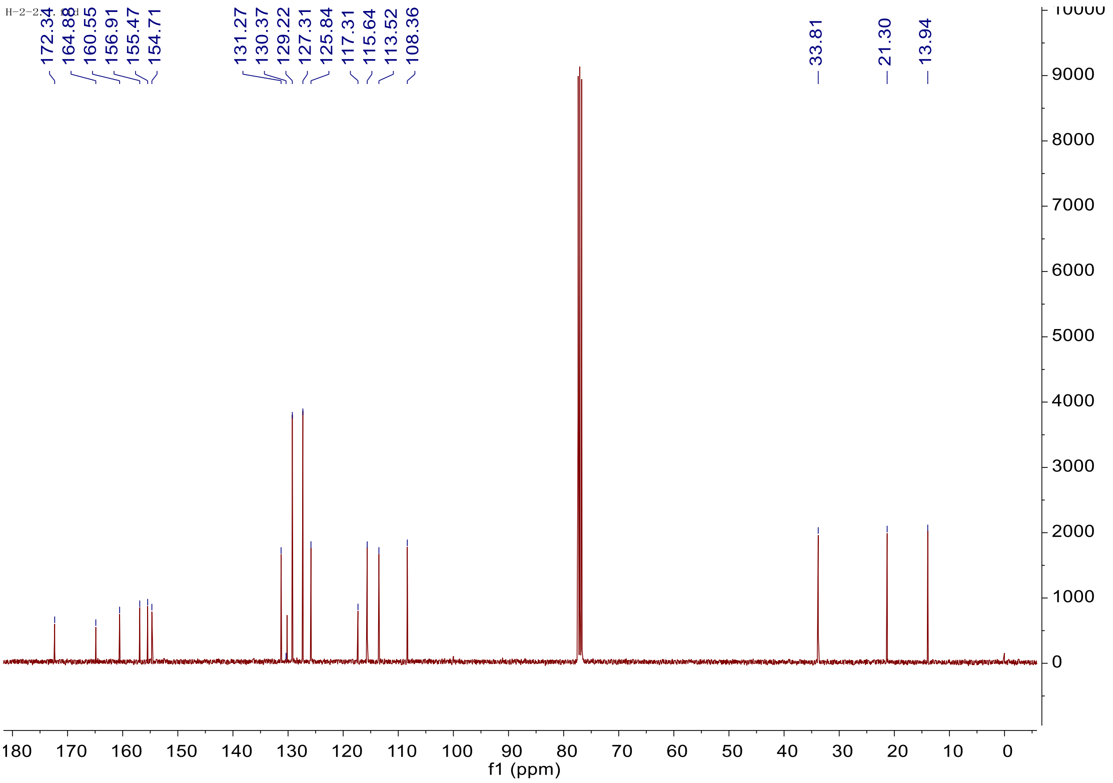

The ^{1}H NMR, ^{13}C NMR spectra of Compound 7c

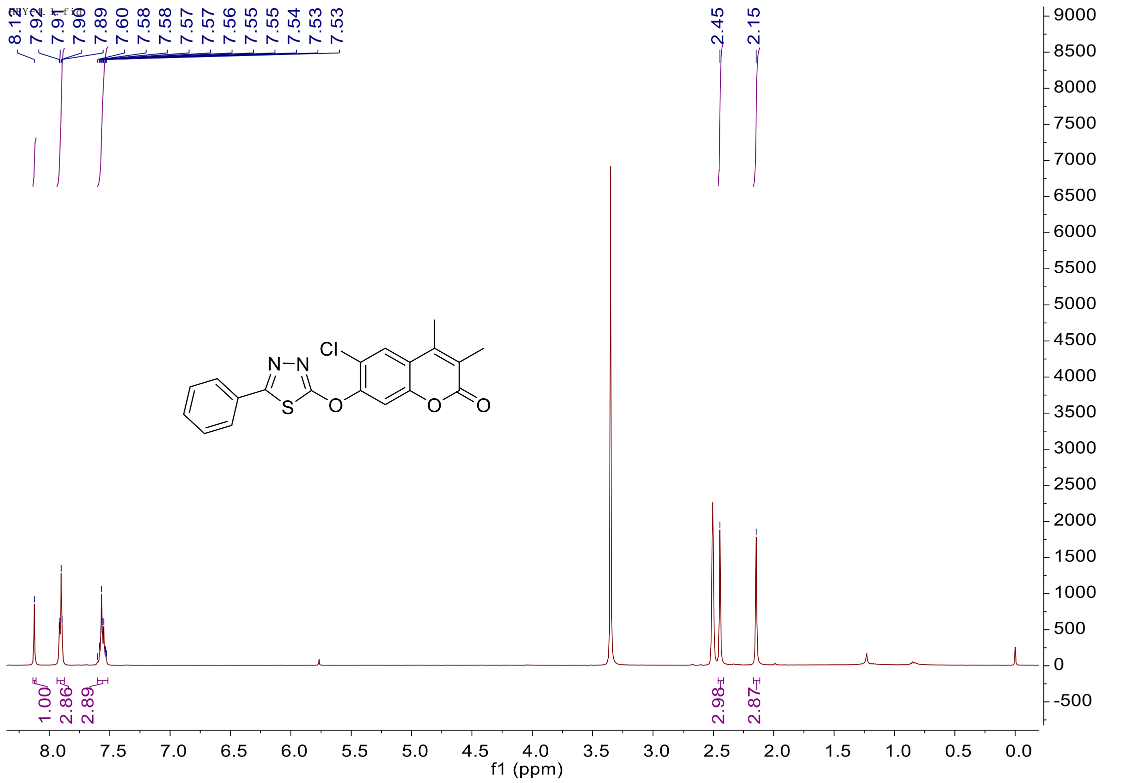

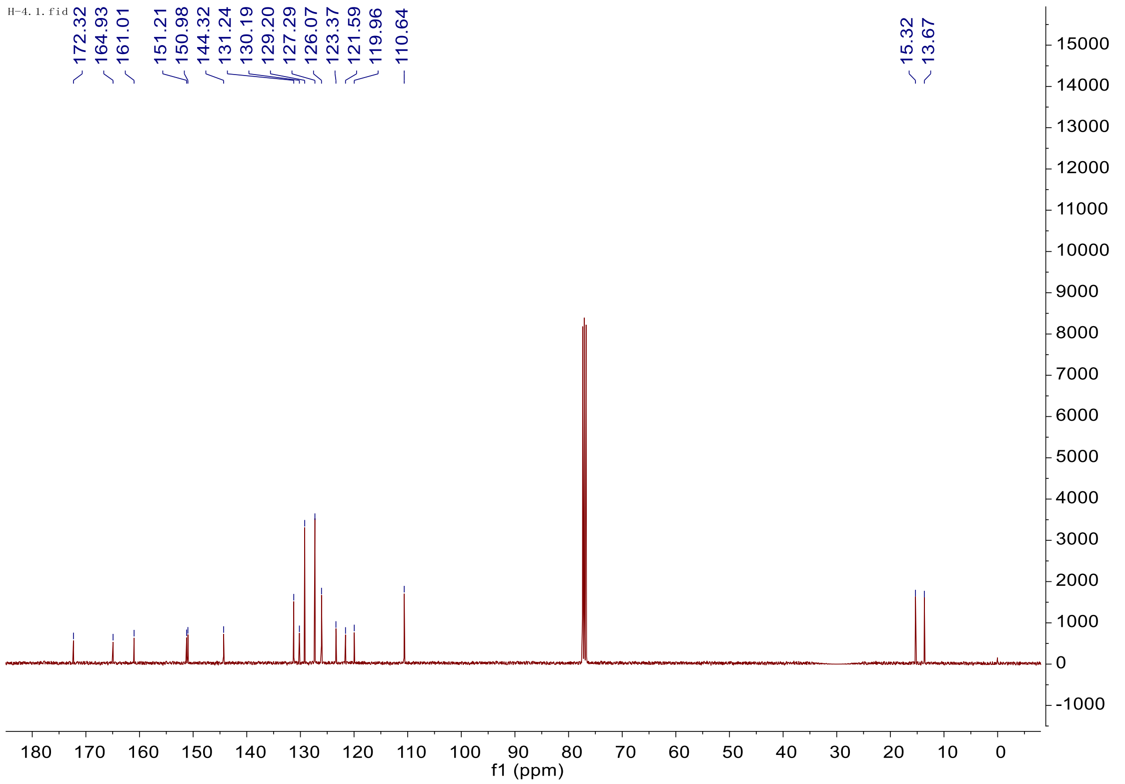

The ^{1}H NMR, ^{13}C NMR spectra of Compound 7g

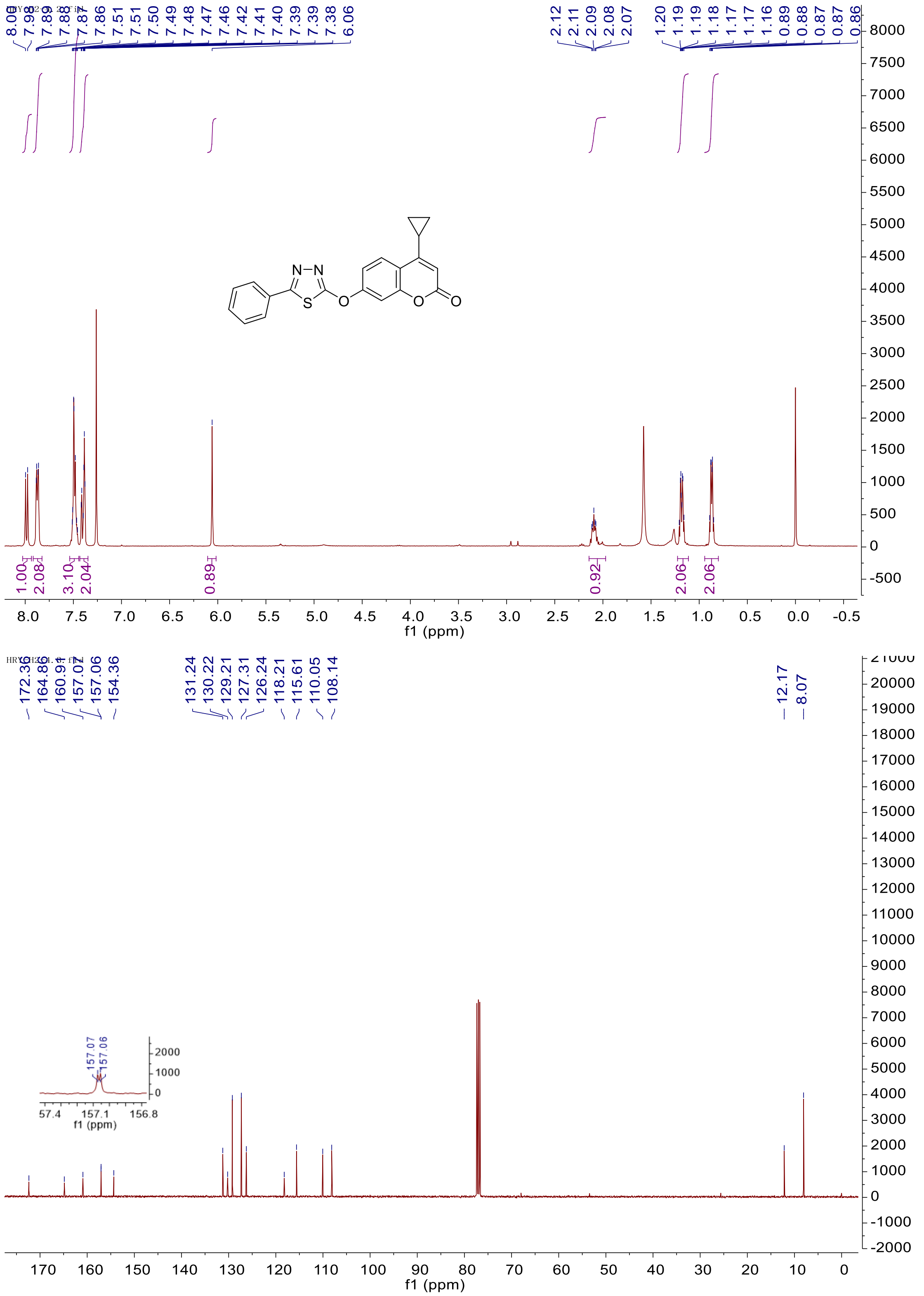

The ^{1}H NMR, ^{13}C NMR spectra of Compound 7k

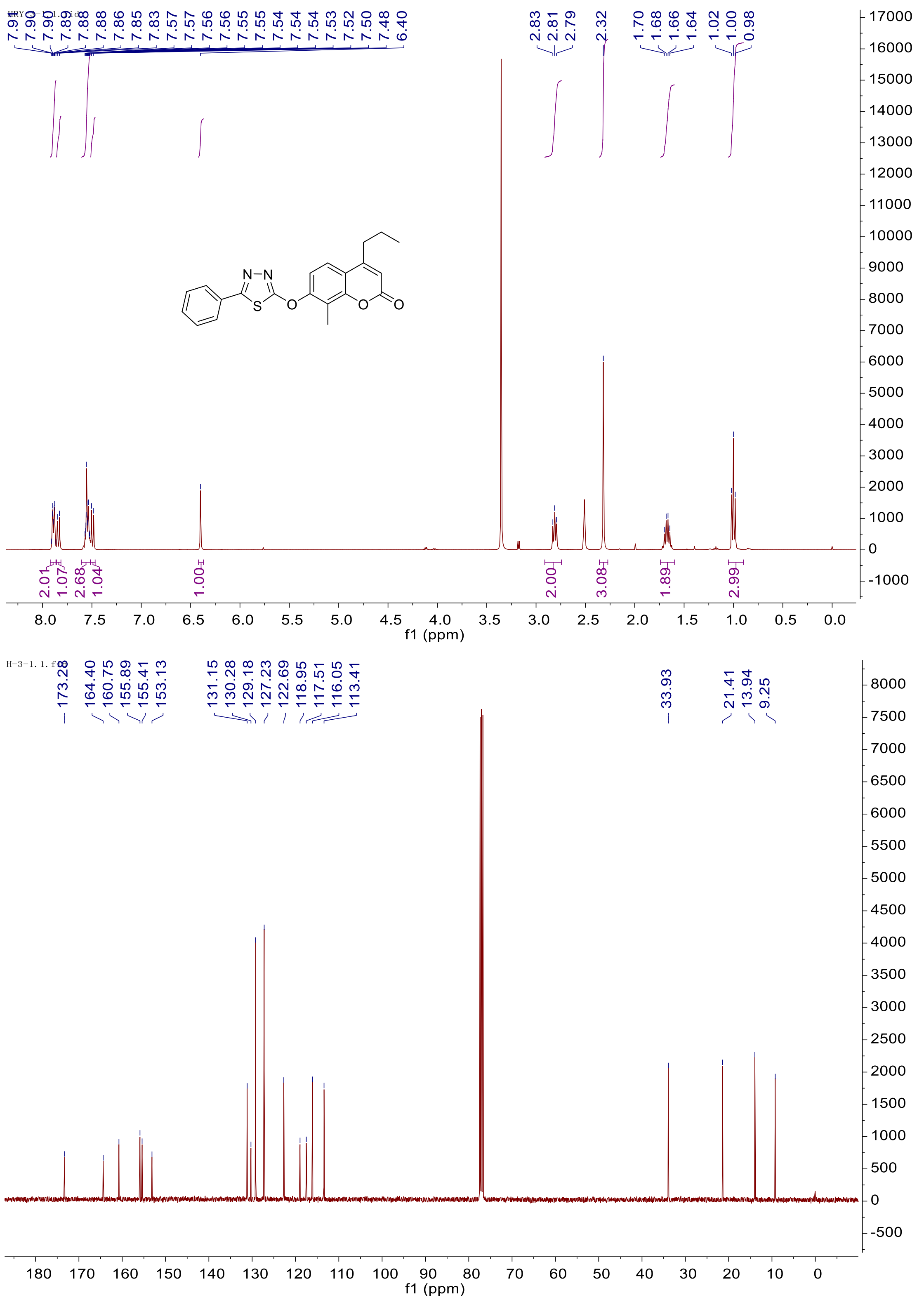

The ^{1}H NMR, ^{13}C NMR spectra of Compound 5a

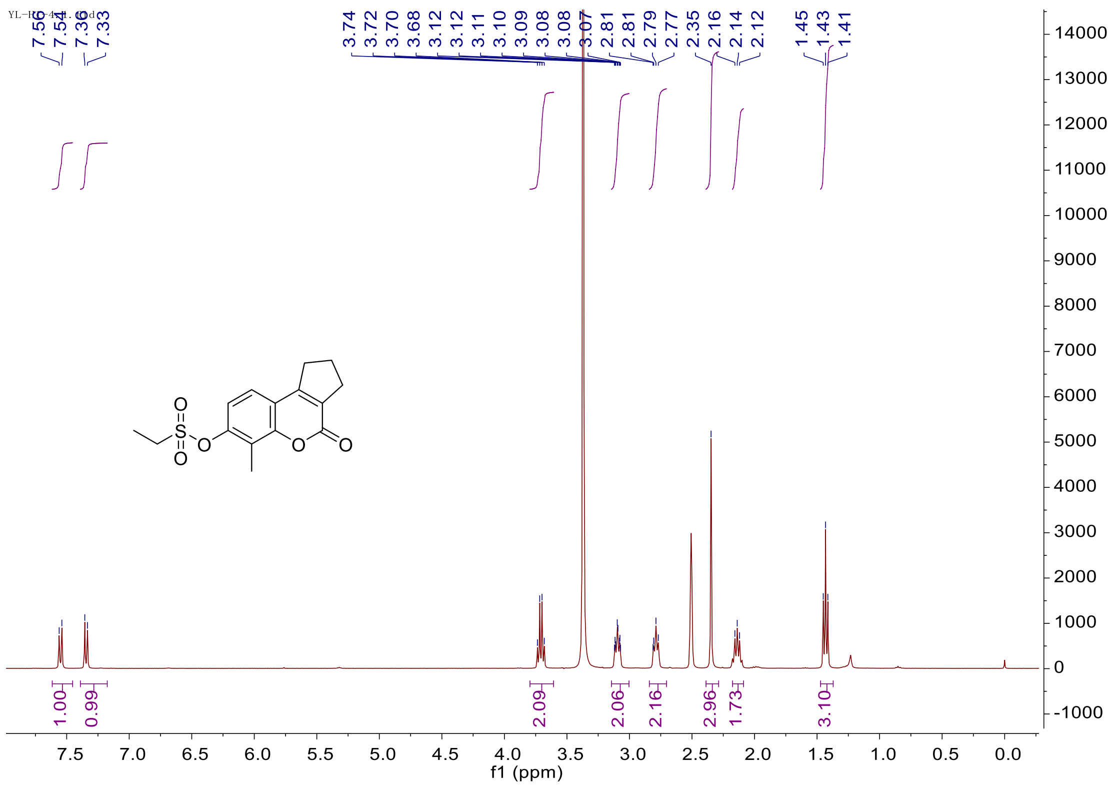

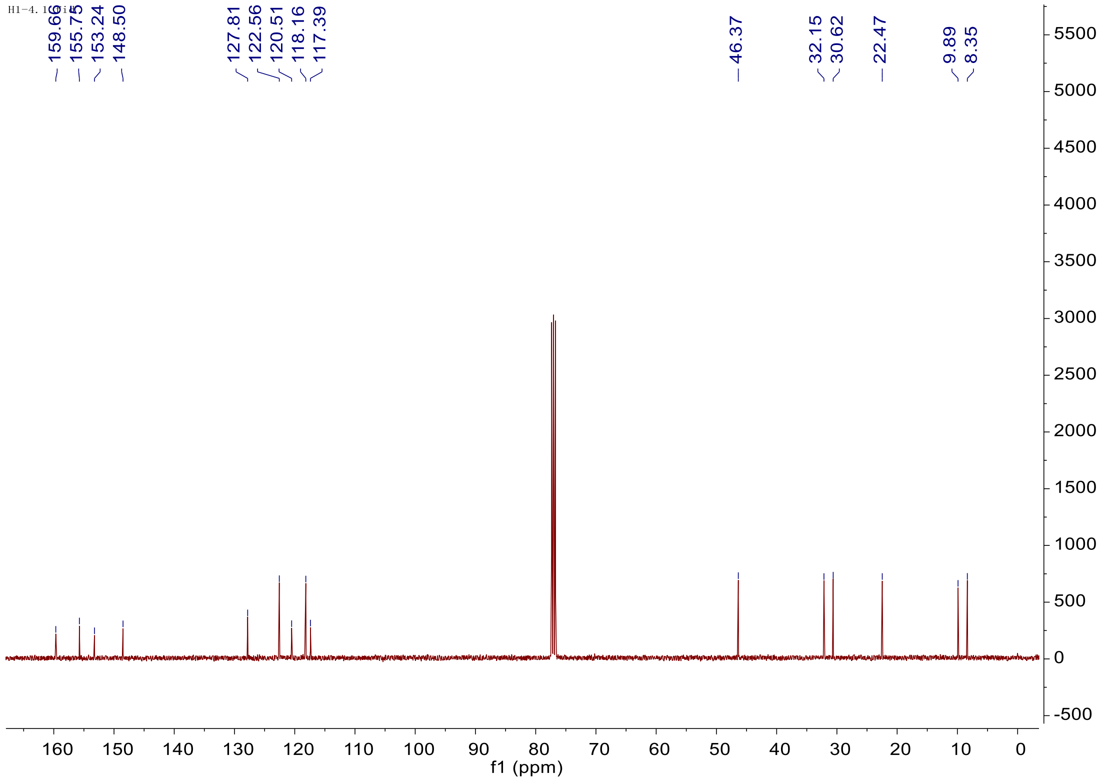

The ^{1}H NMR, ^{13}C NMR spectra of Compound 5b

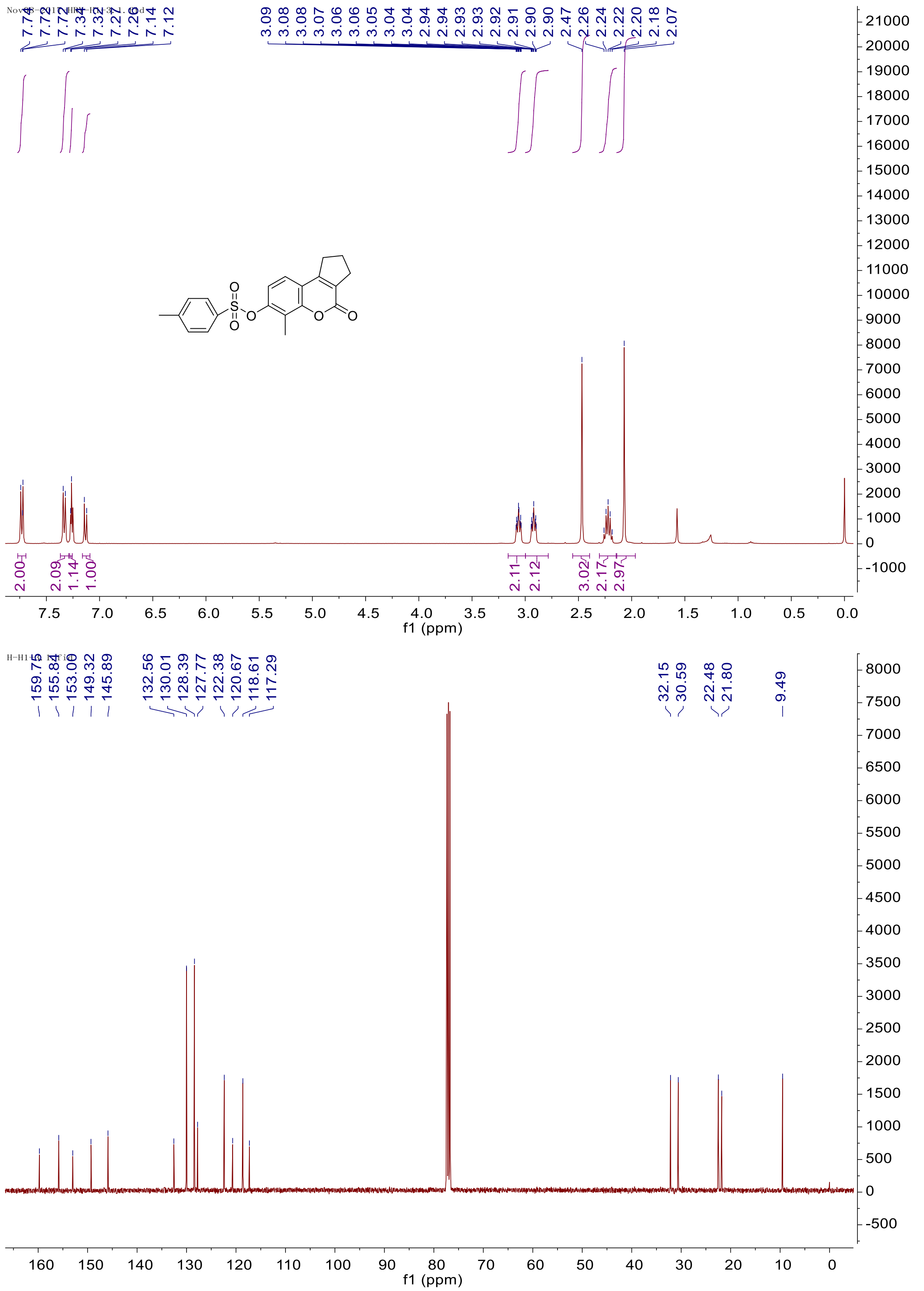

The ^{1}H NMR, ^{13}C NMR spectra of Compound 5c

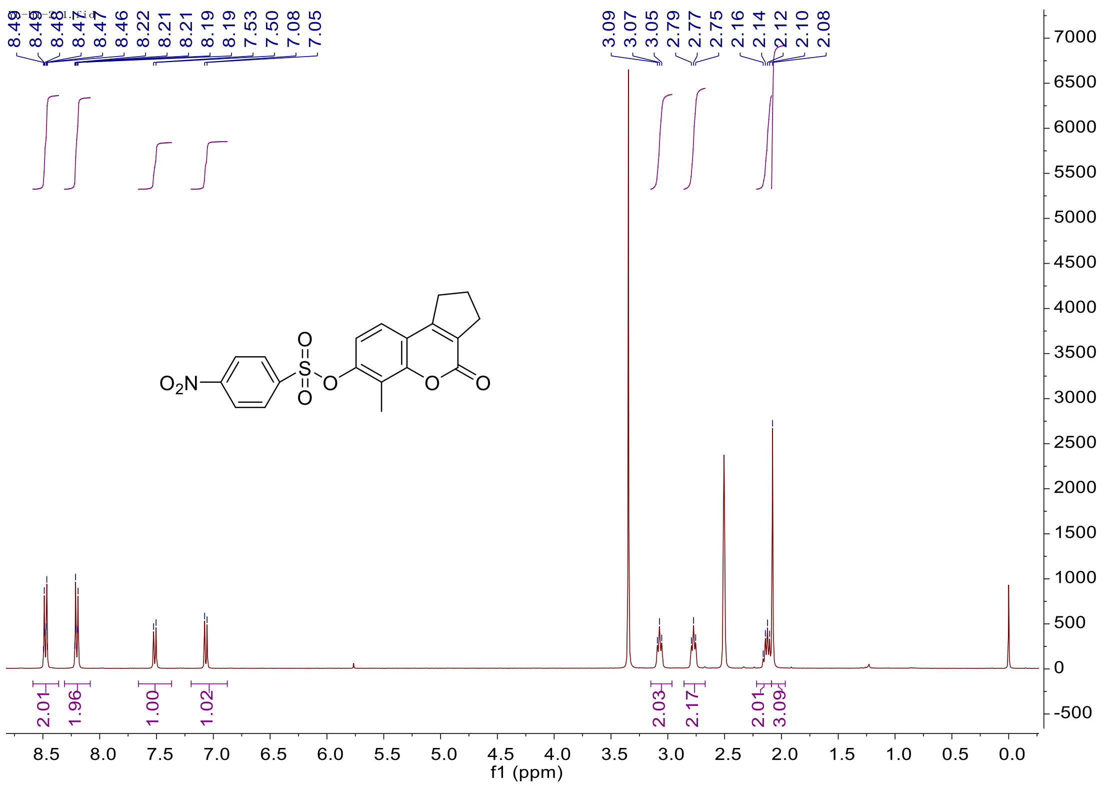

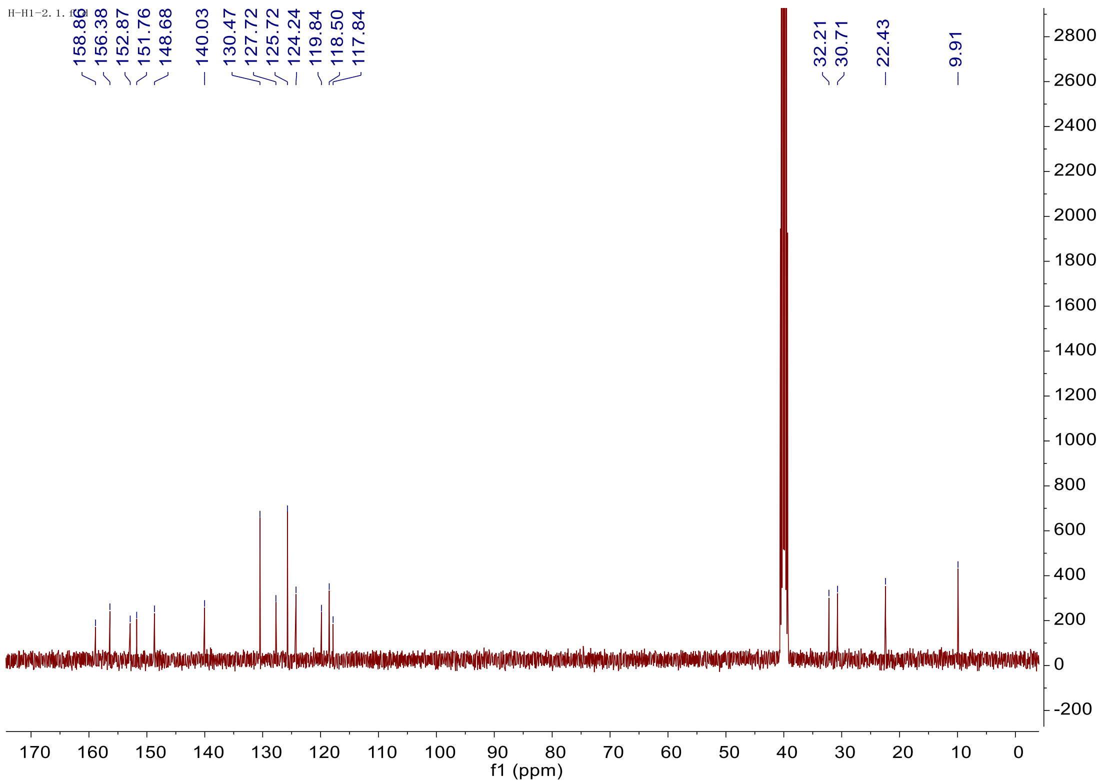

The ^{1}H NMR, ^{13}C NMR spectra of Compound 7d

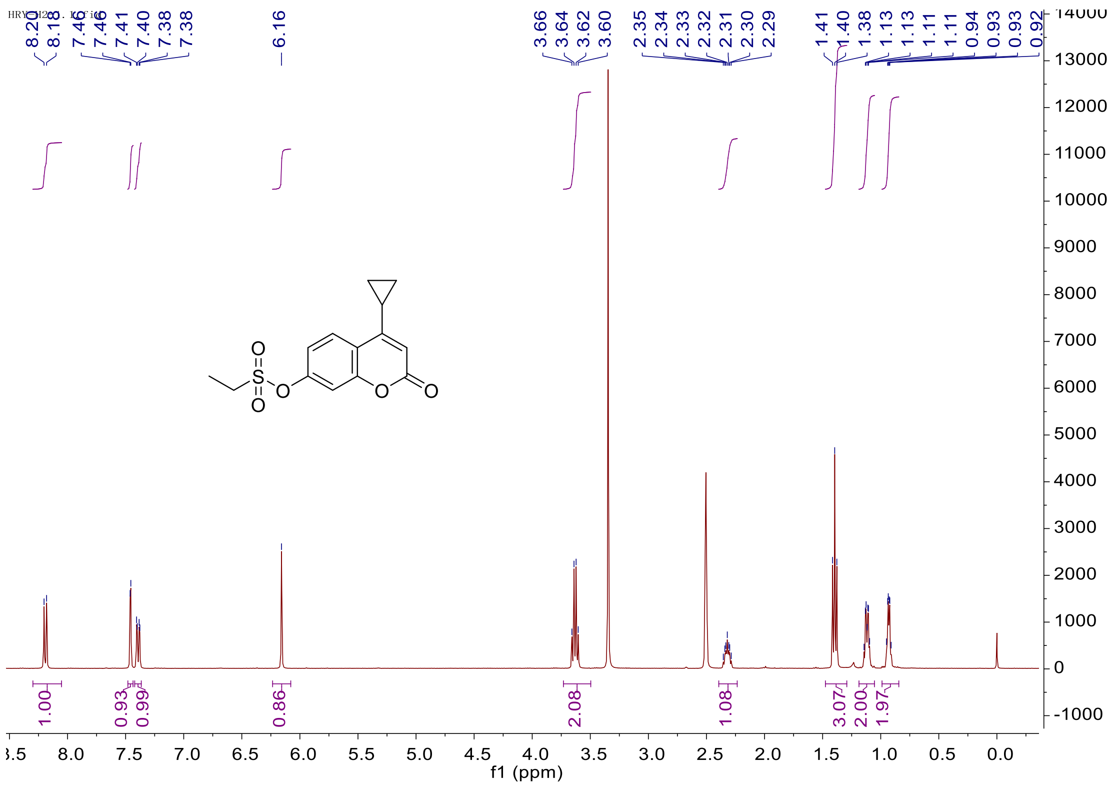

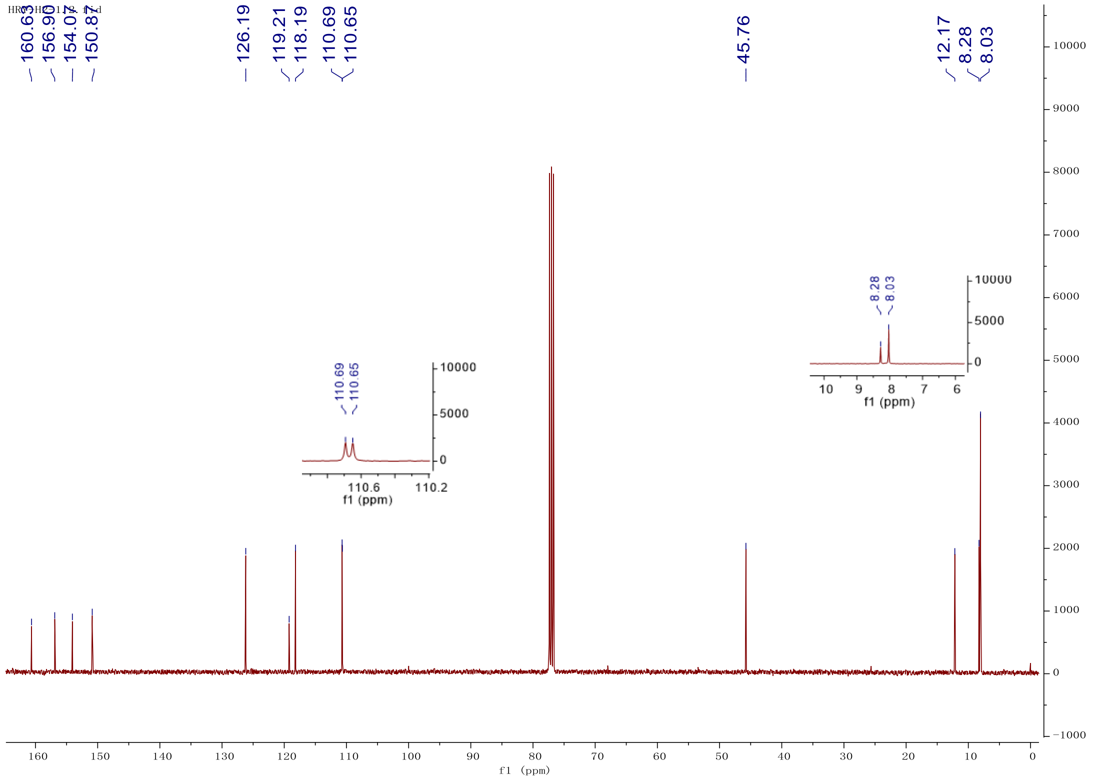

The ^{1}H NMR, ^{13}C NMR spectra of Compound 7e

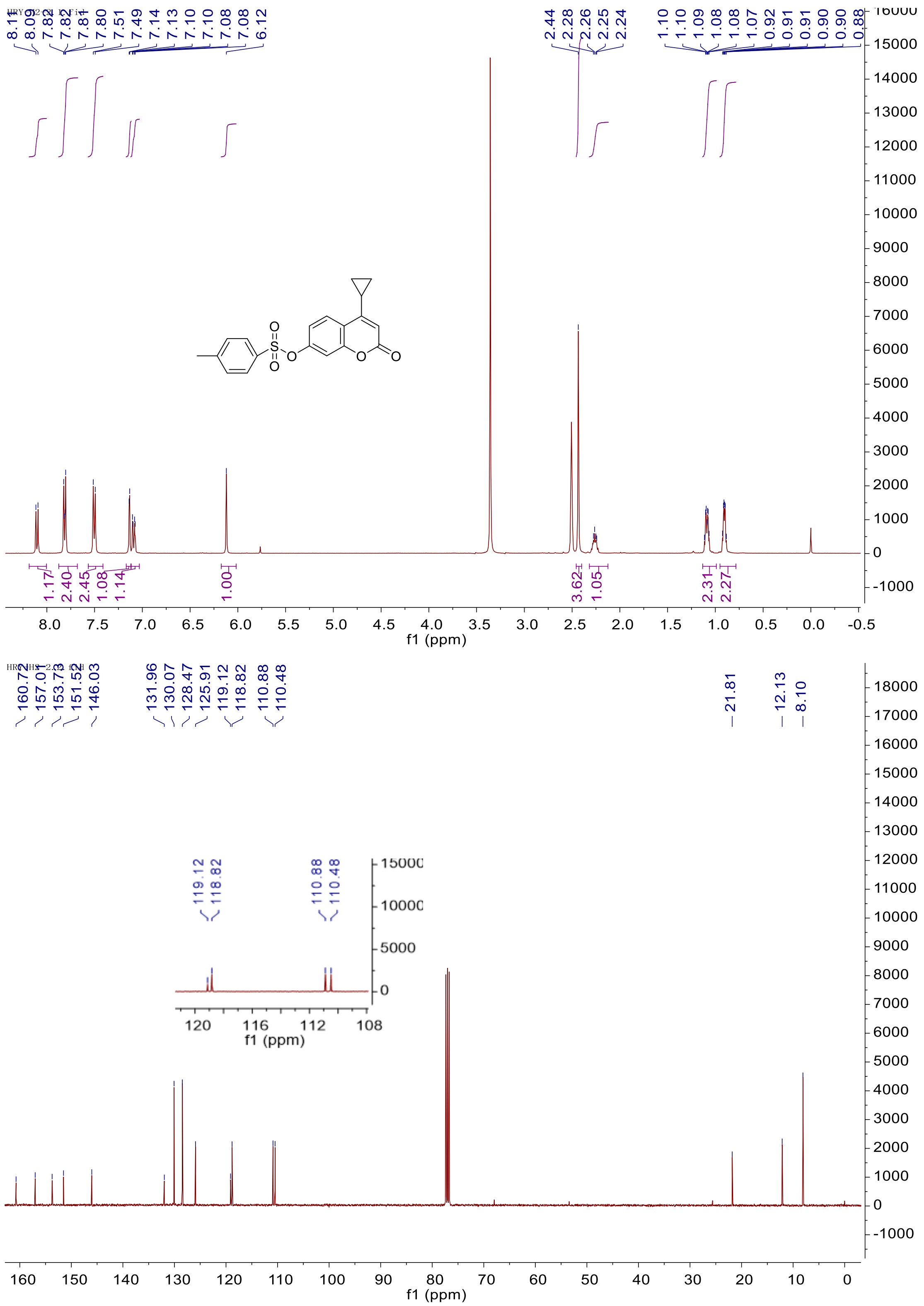

The ^{1}H NMR, ^{13}C NMR spectra of Compound 7f

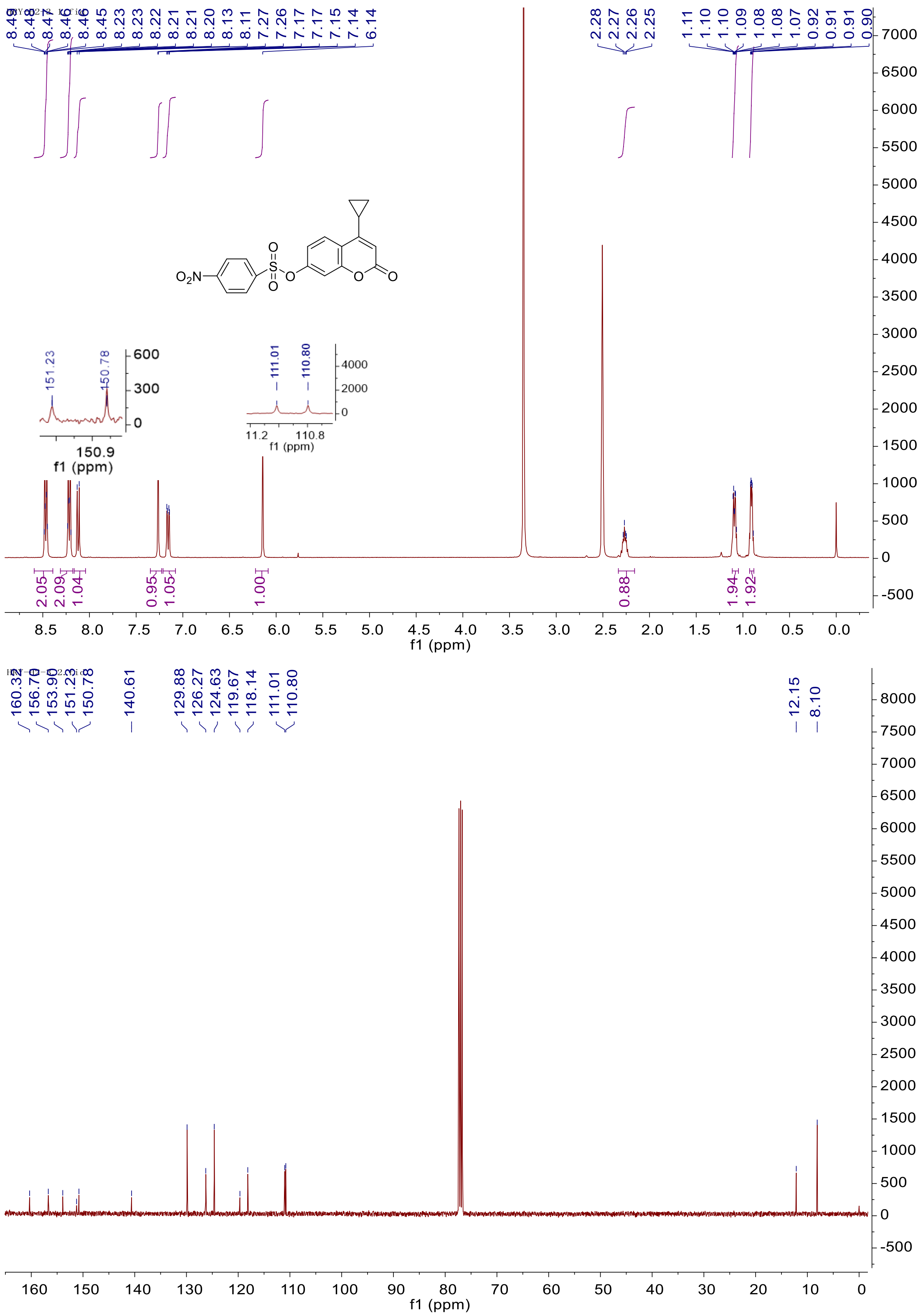

The ^{1}H NMR, ^{13}C NMR spectra of Compound 7h

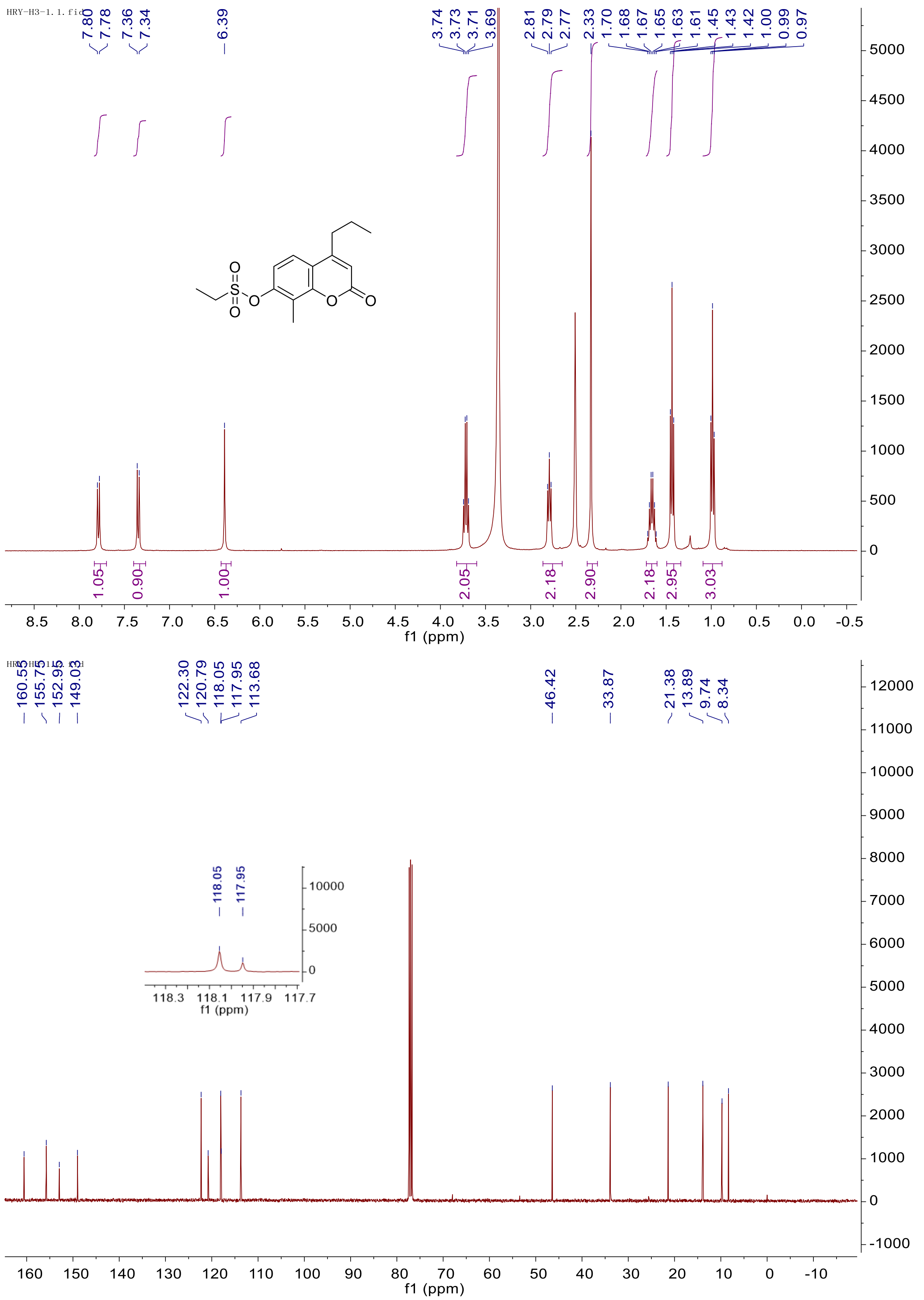

The ^{1}H NMR, ^{13}C NMR spectra of Compound 7i

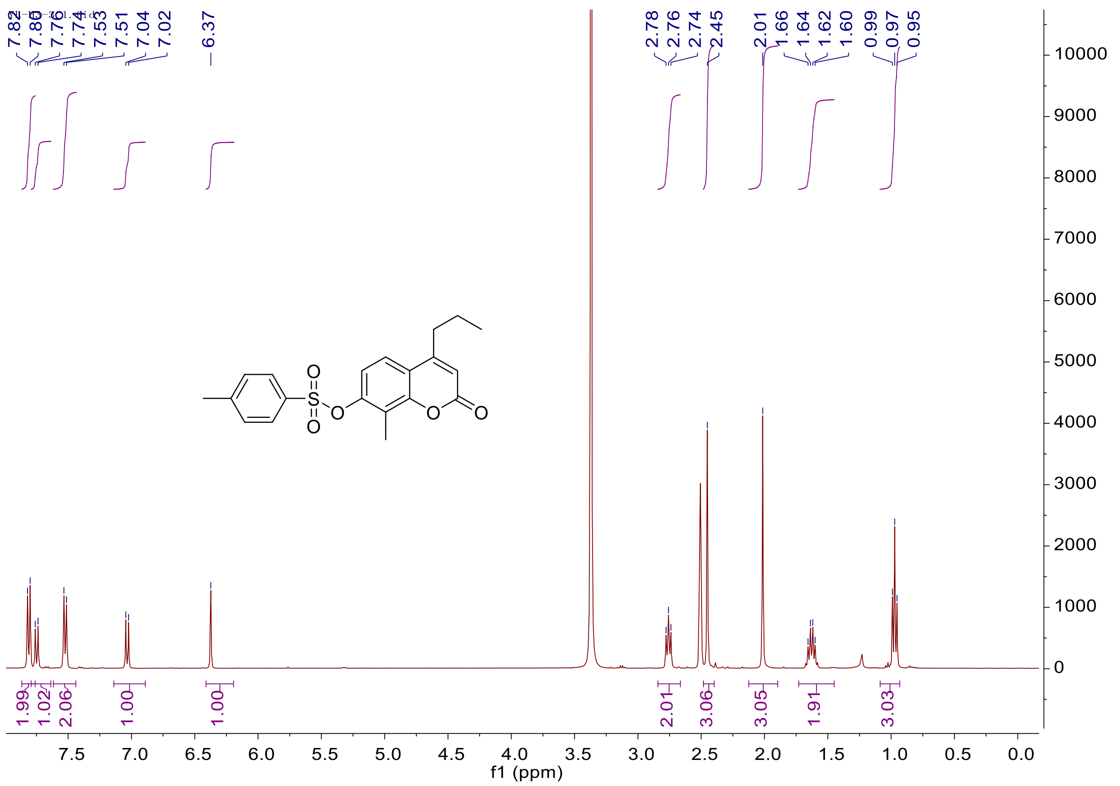

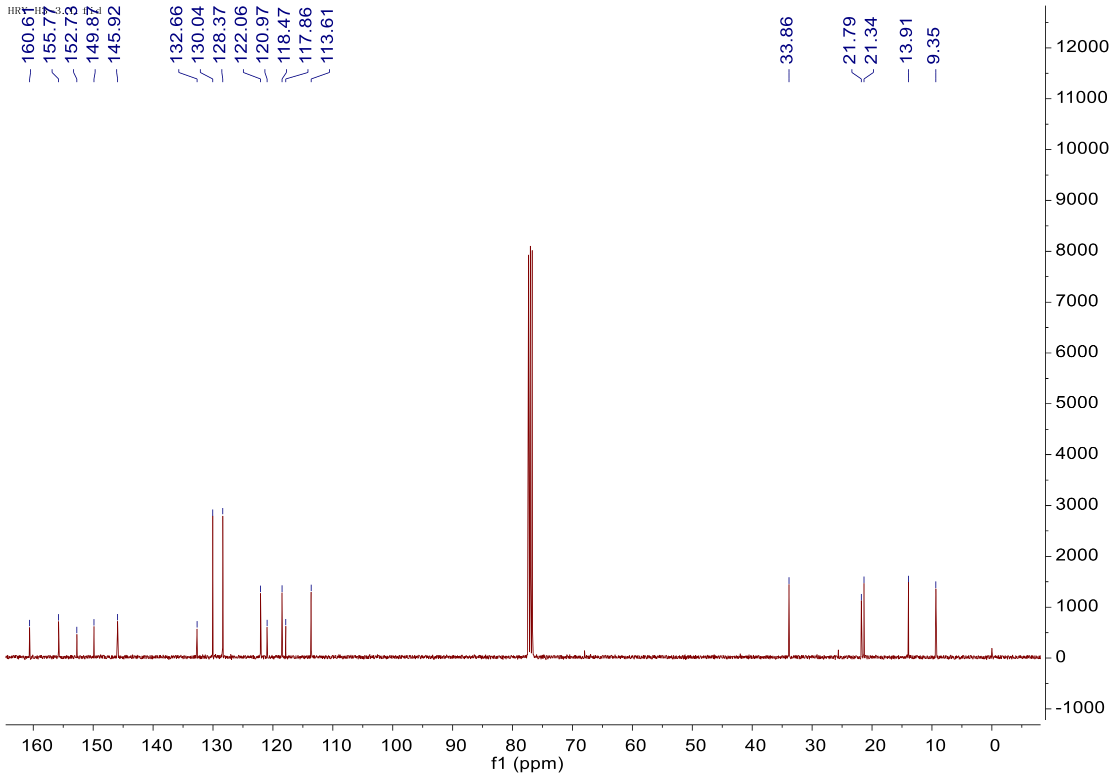

The ^{1}H NMR, ^{13}C NMR spectra of Compound 7j

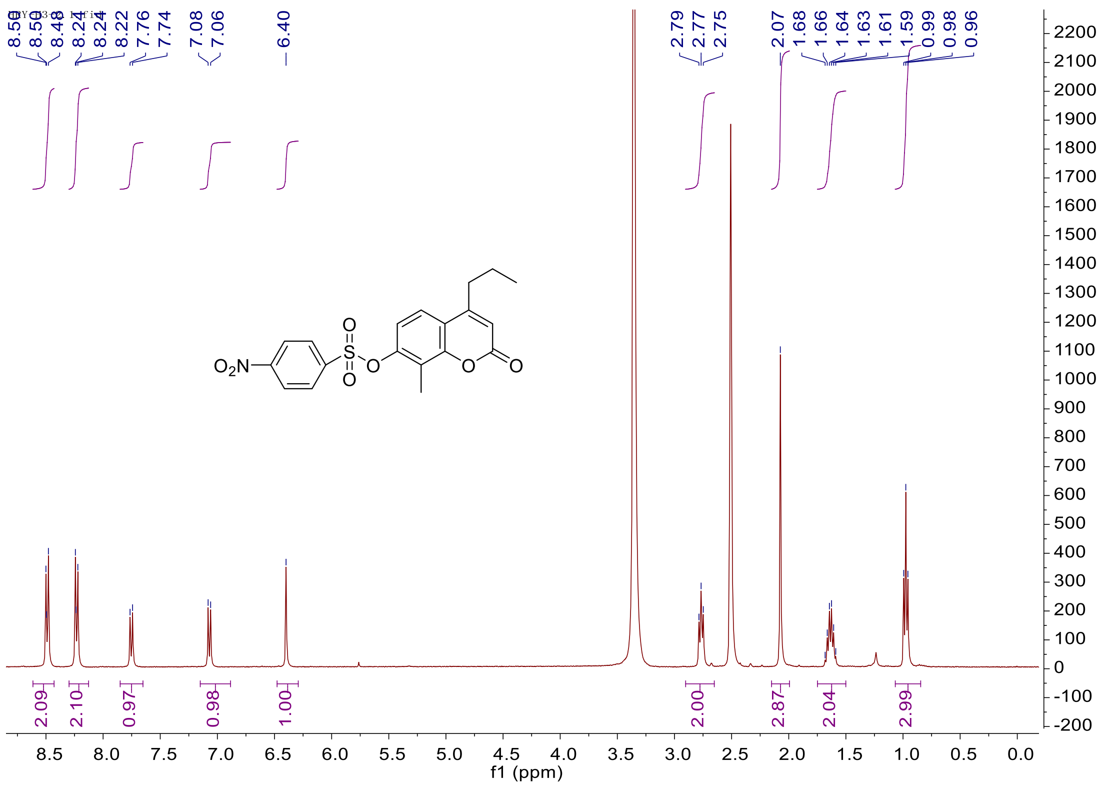
8.50
8.50
8.48
8.24
8.24
8.22
7.76
7.74
7.08
7.06
6.40
2.79
2.77
2.75
2.07
1.68
1.66
1.64
1.63
1.61
1.59
0.99
0.98
0.96
O2N
S
O
O
O
O
O
O
2.09
2.10
0.97
0.98
1.00
2.00
2.87
2.04
2.99
f1 (ppm)

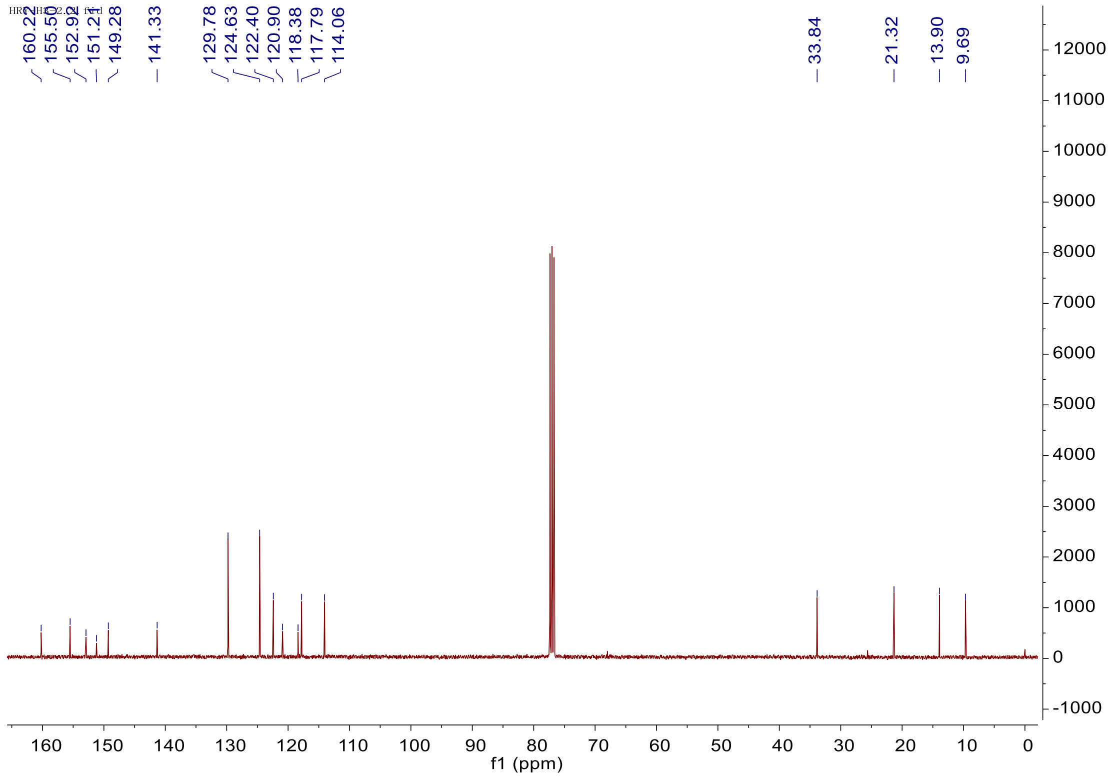
160.22
155.50
152.92
151.24
149.28
141.33
129.78
124.63
122.40
120.90
118.38
117.79
114.06
33.84
21.32
13.90
9.69
f1 (ppm)
